# Highly susceptible SARS-CoV-2 model in CAG promoter–driven hACE2-transgenic mice

**DOI:** 10.1172/jci.insight.152529

**Published:** 2021-10-08

**Authors:** Masamitsu N. Asaka, Daichi Utsumi, Haruhiko Kamada, Satoshi Nagata, Yutaka Nakachi, Tomokazu Yamaguchi, Yoshihiro Kawaoka, Keiji Kuba, Yasuhiro Yasutomi

**Affiliations:** 1 Laboratory of Immunoregulation and Vaccine Research, Tsukuba Primate Research Center,; 2 Laboratory of Biopharmaceutical Research, and; 3 Laboratory of Antibody Design, National Institutes of Biomedical Innovation, Health and Nutrition (NIBIOHN), Ibaraki, Japan.; 4 Global Center for Medical Engineering and Informatics, Osaka University, Suita, Japan.; 5 Department of Molecular Brain Science, Graduate School of Medical Sciences, Kumamoto University, Kumamoto, Japan.; 6 Department of Biochemistry and Metabolic Science, Graduate School of Medicine, Akita University, Akita, Japan.; 7 Influenza Research Institute, Department of Pathobiological Sciences, School of Veterinary Medicine, University of Wisconsin, Madison, USA.; 8 Institute of Medical Science, University of Tokyo, Tokyo, Japan.; 9 National Center for Global Health and Medicine, Tokyo, Japan.

**Keywords:** COVID-19, Mouse models

## Abstract

COVID-19, caused by SARS-CoV-2, has spread worldwide with dire consequences. To urgently investigate the pathogenicity of COVID-19 and develop vaccines and therapeutics, animal models that are highly susceptible to SARS-CoV-2 infection are needed. In the present study, we established an animal model highly susceptible to SARS-CoV-2 via the intratracheal tract infection in CAG promoter–driven human angiotensin-converting enzyme 2–transgenic (CAG-hACE2) mice. The CAG-hACE2 mice showed several severe symptoms of SARS-CoV-2 infection, with definitive weight loss and subsequent death. Acute lung injury with elevated cytokine and chemokine levels was observed at an early stage of infection in CAG-hACE2 mice infected with SARS-CoV-2. Analysis of the hACE2 gene in CAG-hACE2 mice revealed that more than 15 copies of hACE2 genes were integrated in tandem into the mouse genome, supporting the high susceptibility to SARS-CoV-2. In the developed model, immunization with viral antigen or injection of plasma from immunized mice prevented body weight loss and lethality due to infection with SARS-CoV-2. These results indicate that a highly susceptible model of SARS-CoV-2 infection in CAG-hACE2 mice via the intratracheal tract is suitable for evaluating vaccines and therapeutic medicines.

## Introduction

In late December 2019, the SARS-CoV-2, the cause of the COVID-19, was first identified in Wuhan, Hubei province, China, and emerged as a critical pandemic within a few months (1, 2). Infection with SARS-CoV-2 causes asymptomatic or mild respiratory symptoms in most patients, whereas, for patients with risk factors, such as aging and obesity, SARS-CoV-2 induces severe acute lung injury/acute respiratory distress syndrome (ARDS) with high mortality (3). Recently, it has also been reported that SARS-CoV-2 induces not only respiratory symptoms, but also myocarditis, thromboembolism, liver dysfunction, sepsis, and anosmia (4). However, aside from the vaccines, innovative medicine against SARS-CoV-2 infection has not been available, and it will take a long time to for the vaccine to create herd immunity worldwide.

SARS-CoV-2 spike protein interacts with human angiotensin-converting enzyme 2 (hACE2) and enters host cells in vitro and in vivo (5–7). Recently, animal infection models that are susceptible to SARS-CoV-2 have been developed to evaluate vaccines and therapeutic medicines. For instance, nonhuman primates, ferrets, and Syrian hamsters have been used for SARS-CoV-2 infection models (reviewed in ref. 8). These infection models did not show severe symptoms and lethality; therefore, animal models that reflect severe pathological conditions are still needed to evaluate vaccines and therapeutic agents. On the other hand, the mouse is the most used experimental animal because of its simplicity and biological information. However, normal mice are not susceptible to SARS-CoV-2 because of the incompatibility of mouse ACE2 with the SARS-CoV-2 spike protein. Some strategies have been reported for a COVID-19 model in mice expressing hACE2. For instance, transient expression of hACE2 by viral vectors, such as adenovirus (9) and adeno-associated virus (10), in mice with the hACE2 gene knocked in under the endogenous mouse ACE2 promoter (11) and mouse-adapted SARS-CoV2 (12, 13) have been reported, although the symptoms of SARS-CoV-2 were not as severe as in other animal models. Recently, it was reported that K18-hACE2–transgenic mice were infected with SARS-CoV-2 (14–16) and showed body weight loss and lethality with a high dose of SARS-CoV-2 (17–20). These studies mentioned that mouse models that are highly susceptible to SARS-CoV-2 are useful for studying the evaluation of vaccines and therapeutic tools for COVID-19. Hence, animal models of SARS-CoV-2 infection would help develop vaccines and therapeutic agents to resolve the unmet medical needs of the COVID-19 pandemic.

In the present study, we established a SARS-CoV-2 infection model via the respiratory tract using CAG promoter–driven hACE2-transgenic (CAG-hACE2) mice to evaluate therapeutic agents and vaccines. CAG-hACE2 mice showed body weight loss from 5 days after infection (dpi) and died within 10 dpi when infected with 1 × 10^4^ median tissue culture infectious dose (TCID_50_) or 2 × 10^4^ TCID_50_ of SARS-CoV-2. CAG-hACE2 mice were more susceptible to SARS-CoV-2 than previously reported hACE2 mice (17–20). In addition, time-dependent viral replication, lung injury with infiltration of inflammatory cells, and elevated cytokine and chemokine levels in the lungs infected with SARS-CoV-2 were also explored. Moreover, at least 15 hACE2 genes were integrated into chromosome 1 of CAG-hACE2 mice, and hACE2 was highly expressed in the lung and heart. Most importantly, the protection efficacy against SARS-CoV-2 by immunization with receptor-binding domain (RBD) protein and injection of plasma prepared from RBD-immunized mice were also evaluated in this model. These results indicate that the SARS-CoV-2 infection model with CAG-hACE2 mice is beneficial for the evaluation of vaccine development and screening of medical products for COVID-19.

## Results

### SARS-CoV-2 intratracheal infection in CAG-hACE2 mice.

**Figure 1:**
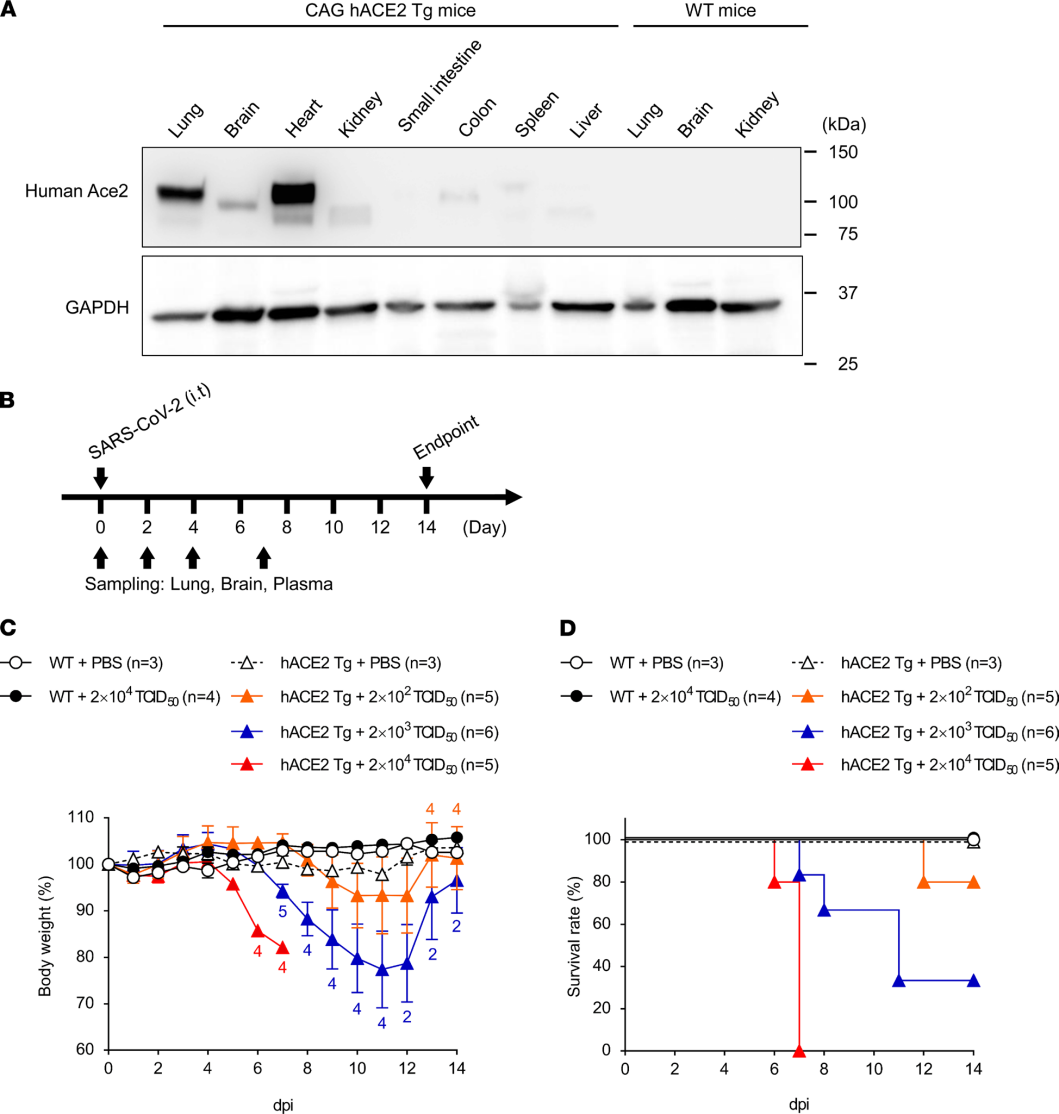
The pathogenesis of SARS-CoV-2 infection is exacerbated in a viral dose-dependent manner. (**A**) Western blot of human ACE2 protein using various organs in WT and CAG-hACE2 mice. The top and bottom rows show hACE2 and GAPDH, respectively. (**B**) Schematic diagram of experimental schedule. Male and female C57BL/6 and CAG-hACE2 mice were infected via respiratory tract with SARS-CoV-2 (2 × 10^2^ TCID_50_, *n* = 5; 2 × 10^3^ TCID_50_, *n* = 6; 2 × 10^4^ TCID_50_, *n* = 5) and were administrated with an equal volume of PBS for mock infection controls (WT, *n* = 3; CAG-hACE2 mice, *n* = 3). Body weight and survival were recorded daily for up to 14 days. (**C** and **D**) Percentage of initial body weight (**C**) and survival rate (**D**). Numbers in **C** represent the number of mice measured for body weight at each time. White and black circles indicate WT with mock infection and WT infected with 2 × 10^4^ TCID_50_, respectively. Triangles denote CAG-hACE2 mice. White, orange, blue, and red triangles represent PBS, 2 × 10^2^ TCID_50_, 2 × 10^3^ TCID_50_, and 2 × 10^4^ TCID_50_, respectively.

First, the expression of hACE2 in CAG-hACE2 mice was examined by Western blotting. The expression levels of hACE2 protein in CAG-hACE2 mice were marked higher in the lung and heart and were also detected in the brain. On the other hand, hACE2 expression was detected or not detected in the kidney, small intestine, colon, and spleen (Figure 1A). Next, CAG-hACE2 mice were intranasally infected with SARS-CoV-2 at doses of 2 × 10^2^ TCID_50_ (low dose, *n* = 6), 2 × 10^3^ TCID_50_ (middle dose, *n* = 6), and 2 × 10^4^ TCID_50_ (high dose, *n* = 6), and body weight and survival rates were recorded for up to 14 days (Supplemental Figure 1; supplemental material available online with this article; https://doi.org/10.1172/jci.insight.152529DS1). Most CAG-hACE2 mice died, even with low viral titers, suggesting the notion that CAG-hACE2 mice are highly susceptible to SARS-CoV-2. However, the mortality of intranasally infected CAG-hAVE2 mice was not dose dependent. Previously, Hong et al. have reported that intratracheal infection improved infection efficiency and evaluated the efficacy of dexamethasone against SARS-CoV-2 infection (21). We also tried to establish infection models using CAG-hACE2 mice via intratracheal infection with SARS-CoV-2 to evaluate lung injury, such as pneumonia, and the efficacy of vaccines and therapeutic agents against SARS-CoV-2 infection. To determine the susceptibility and lethality of CAG-hACE2 mice intratracheally infected with SARS-CoV-2, C57BL/6 and CAG-hACE2 mice were infected with doses of 2 × 10^2^ TCID_50_ (low dose, *n* = 5), 2 × 10^3^ TCID_50_ (middle dose, *n* = 6), and 2 × 10^4^ TCID_50_ (high dose, *n* = 5), and body weight and survival rates were recorded for up to 14 days (Figure 1B). CAG-hACE2 mice infected with the high and middle viral doses showed body weight loss at 5 and 7 dpi, respectively (Figure 1C). CAG-hACE2 mice infected with the low viral dose showed a reduction in body weight starting from 9 dpi and recovered at 12 dpi. Mortality was significantly elevated in infected CAG-hACE2 mice in a dose-dependent manner (Figure 1D). All 5 CAG-hACE2 mice infected with the high viral dose died within 7 dpi, and 4 of the 6 CAG-hACE2 mice infected with the middle viral dose died. Four of the five CAG-hACE2 mice infected with a low viral dose did not show fatal results for up to 14 days. It is noteworthy that the lethal titer of SARS-CoV-2 in CAG-hACE2 mice was lower than that in other transgenic mice (17–20). PBS-treated WT mice (*n* = 3), PBS-treated CAG-hACE2 mice (*n* = 3), and WT mice infected with the high viral dose (*n* = 4) did not show weight loss, and all these mice were alive during the experimental period. The tissue distributions of the viral genome were predominantly in the lung and brain, with a slight presence in the heart, kidney, spleen, small intestine, and colon (Supplemental Figure 2). The viral titers in the lungs and brains of CAG-hACE2 mice, which were intratracheally infected with SARS-CoV-2, were dose dependent (Supplemental Figure 2). These results demonstrate that CAG-hACE2 mice are highly susceptible to SARS-CoV-2, and the lungs and brain are the main targets of SARS-CoV2 infection.

### Levels of SARS-CoV2 infection in the lungs and brain of CAG-hACE2 mice.

**Figure 2:**
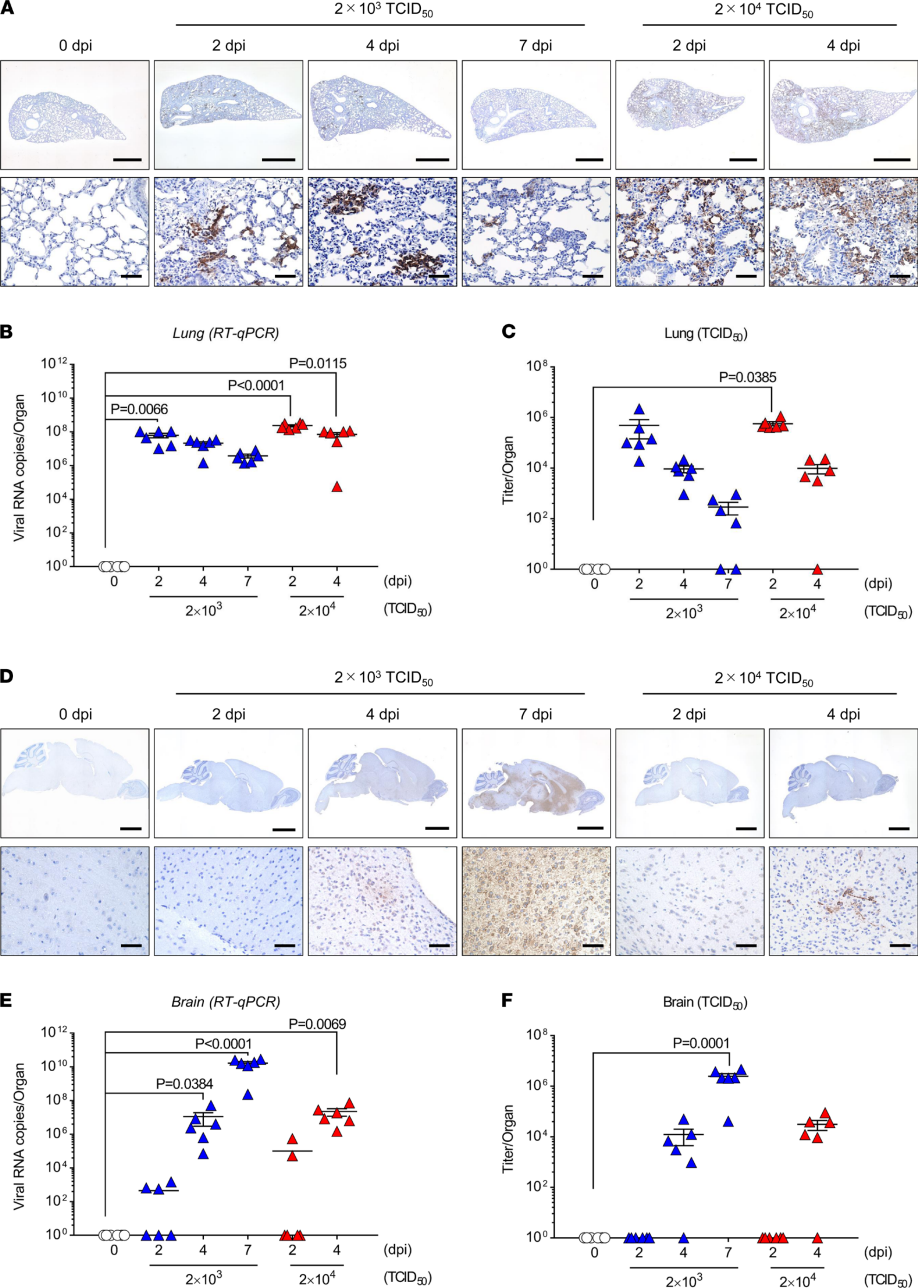
Immunohistological staining and virus titer of SARS-CoV-2 infection in CAG-hACE2 mice. (**A** and **D**) Nucleocapsid protein of SARS-CoV-2 in lung (**A**) and brain (**D**) sections from CAG-hACE2 mice at 0, 2, 4, and 7 days after infection (dpi). Scale bars: 50 μm (bottom rows); 1000 μm (top row, **A**); 2000 μm (top row, **D**). (**B**, **C**, **E**, and **F**) qRT-PCR of SARS-CoV-2 N gene expression (**B** and **E**) and TCID_50_ (**C** and **F**) in the lung and brain tissue at 0, 2, 4 and 7 dpi. Blue and red triangles indicate infection doses of 2 × 10^3^ TCID_50_ and 2 × 10^4^ TCID_50_, respectively. Data are presented as the mean ± SEM. Statistical analyses were performed using Kruskal-Wallis 1-way ANOVA followed by Dunn’s multiple comparison test. All groups, *n* = 6 mice.

Based on data in Supplemental Figure 2, we focused on the lung and brain and evaluated the viral infection levels at 0, 2, 4, and 7 dpi (*n* = 6 per time point). To examine the infection levels of SARS-CoV-2, the nucleocapsid protein (N protein) of SARS-CoV-2 in the lung and brain sections was detected by immunohistochemistry (Figure 2, A and D). The expression of SARS-CoV-2 N protein in the lung, which was infected with the middle viral dose, was mainly localized to alveolar epithelial cells and diffused throughout the lung section at 2 dpi. The expression of N protein was also observed at 4 and 7 dpi and then gradually decreased. The expression of N protein in the lungs of mice administered high and middle viral doses was localized to alveolar epithelial cells; however, the expression levels of N protein in the lungs infected with the high viral dose were much higher than those in lungs with middle dose. On the other hand, the expression of SARS-CoV-2 N protein in the brain was restricted to a few areas at 4 dpi in mice administered both the middle and high viral doses. The expression of N protein was high and diffused throughout the brain section infected with the middle dose at 7 dpi. To confirm immunohistochemical analysis more quantitatively, we evaluated SARS-CoV-2 infection levels using RT-qPCR (Figure 2, B and C) and TCID_50_ assays (Figure 2, E and F). The viral RNA copies and viral titers in the lung were significantly elevated at 2 dpi, and these titers gradually decreased at 4 and 7 dpi. In contrast, the viral RNA copies and viral titers in the brain increased over time, and the results of RT-qPCR and TCID_50_ assays were consistent with those of immunohistostaining. These results suggest that SARS-CoV-2 intratracheally administered to CAG-hACE2 mice initially infected the lung and then gradually transmitted to the brain.

### Histological changes in the lungs of SARS-CoV-2–infected CAG-hACE2 mice.

**Figure 3:**
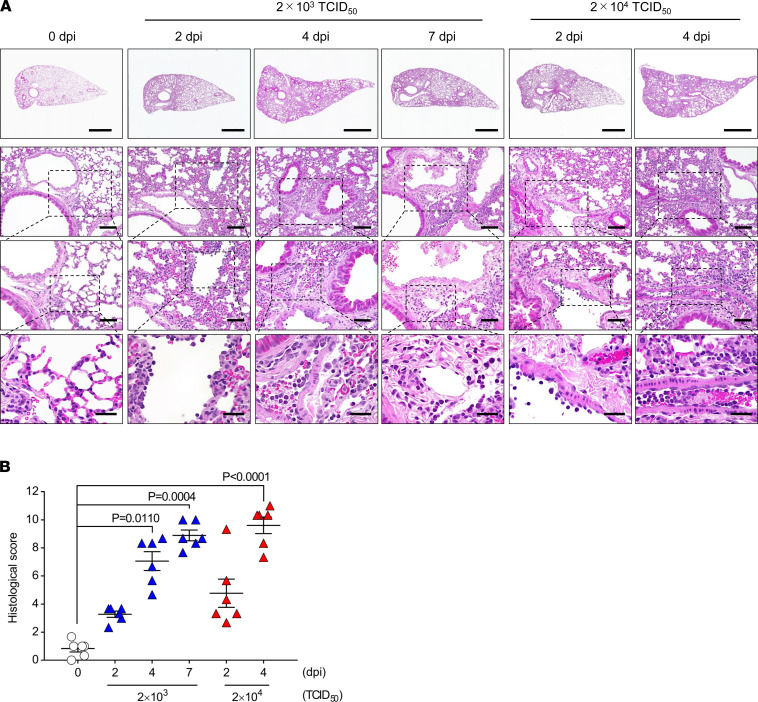
Evaluation of histopathology in lung injury after SARS-CoV-2 infection. (**A**) H&E staining of representative images in lung tissues at 0, 2, 4, and 7 days after infection (dpi). Scale bars: 1000 μm (top row); 100 μm (second row); 50 μm (third row); 25 μm (bottom row). (**B**) Score of lung injuries at 0, 2, 4, and 7 dpi. White triangles indicate mock infections. Blue and red triangles represent infection doses of 2 × 10^3^ TCID_50_ and 2 × 10^4^ TCID_50_, respectively. Data are presented as the mean ± SEM. Statistical analyses were performed using the Kruskal-Wallis 1-way ANOVA followed by Dunn’s multiple comparison test. All groups, *n* = 6 mice.

The aggravation of pulmonary injury in CAG-hACE2 mice infected with SARS-CoV-2 was analyzed by H&E staining, and increasing histological scores were observed in a time-dependent manner (Figure 3). The infiltration of inflammatory cells, mainly neutrophils and lymphocytes, was observed around the bronchioles at 2 dpi in lungs infected with both middle and high doses. Alveolar wall thickening was observed at 4 dpi, and the inflammatory cells infiltrated the adjacent interstitial spaces. The histological changes in CAG-hACE2 mice infected with middle viral doses were observed at similar levels of lung injury at 7 and 4 dpi. In addition, severe pneumonia and alveolar wall thickening with progressive infiltration of inflammatory cells were observed in the lungs infected with high viral doses. Severe alveolar congestion and/or hemorrhage was observed in the middle dose–infected CAG-hACE2 mice. In the low dose–infected CAG-hACE2 mice, slight infiltration of inflammatory cells and exudate was observed (Supplemental Figure 3). These data indicated that SARS-CoV-2 infection in CAG-hACE2 mice induced serious acute lung injury with a high level of inflammatory cell infiltration.

### Inflammatory responses to SARS-CoV-2 infection in CAG-hACE2 mice.

**Figure 4:**
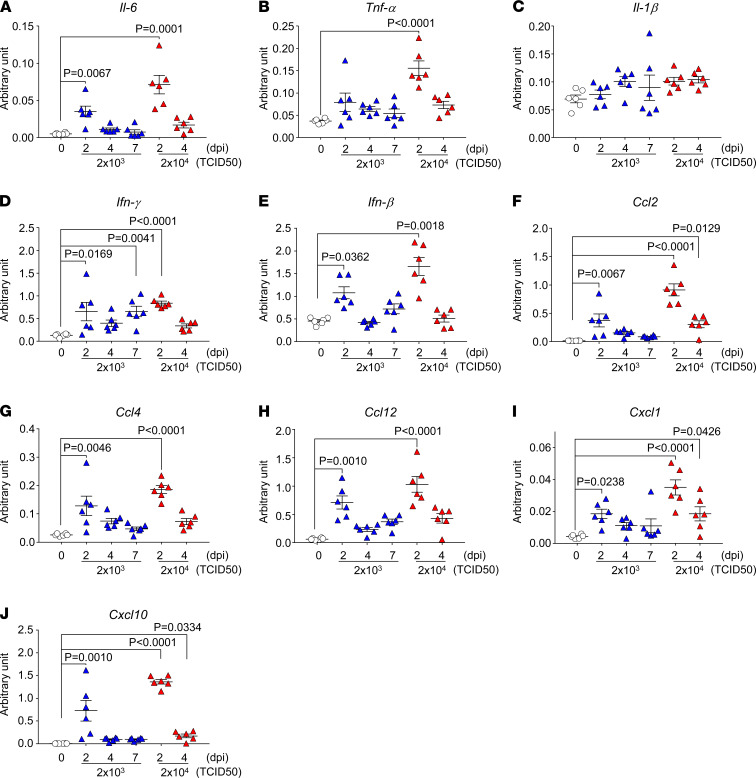
mRNA levels of cytokines and chemokines in the lungs of CAG-hACE2 mice infected with SARS-CoV-2. Expression levels of cytokines and chemokines in lung. mRNA expression of *Il-6* (**A**), *Tnf-*α (**B**), *Il-1*β (**C**), *Ifn-*γ (**D**), *Ifn-*β (**E**), *Ccl2* (**F**), *Ccl4* (**G**), *Ccl12* (**H**), *Cxcl1* (**I**) and *Cxcl10* (**J**) was normalized with that of β*-actin* mRNA at 0, 2, 4, and 7 days after infection. White triangles indicate the mock infection. Blue and red triangles represent infection doses of 2 × 10^3^ TCID_50_ and 2 × 10^4^ TCID_50_, respectively. Data are presented as the mean ± SEM. Statistical analyses were performed using Kruskal-Wallis 1-way ANOVA followed by Dunn’s multiple comparison test. All groups, *n* = 6 mice.

**Figure 5:**
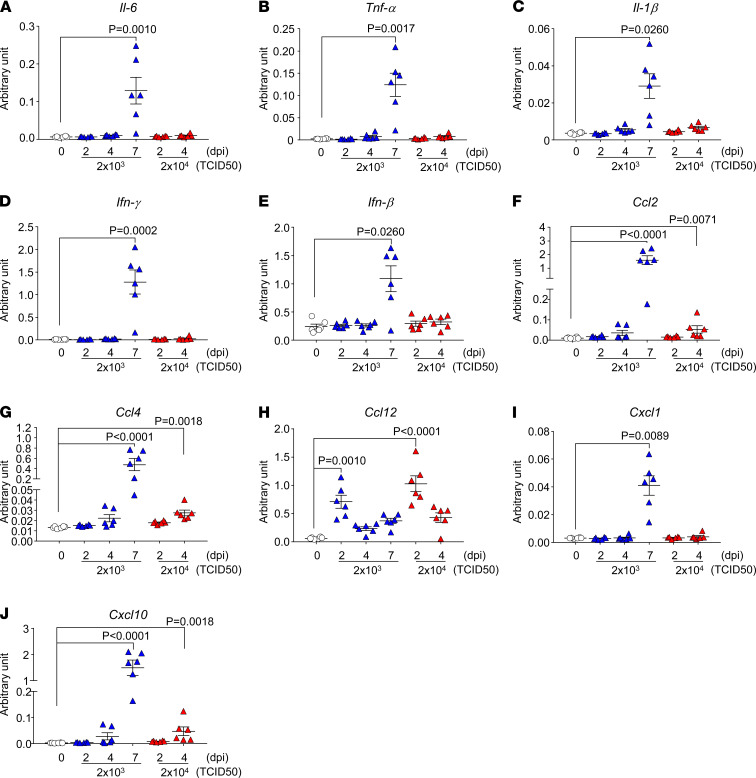
mRNA levels of cytokines and chemokines in the brains of CAG-hACE2 mice infected with SARS-CoV-2. Expression levels of cytokines and chemokines in brain. mRNA expression of *Il-6* (**A**), *Tnf-*α (**B**), *Il-1*β (**C**), *Ifn-*γ (**D**), *Ifn-*β (**E**), *Ccl2* (**F**), *Ccl4* (**G**), *Ccl12* (**H**), *Cxcl1* (**I**) and *Cxcl10* (**J**) was normalized with that of β*-actin* mRNA at 0, 2, 4, and 7 days after infection. White triangles indicate the mock infection. Blue and red triangles represent infection doses of 2 × 10^3^ TCID_50_ and 2 × 10^4^ TCID_50_, respectively. Data are presented as the mean ± SEM. Statistical analyses were performed using Kruskal-Wallis 1-way ANOVA followed by Dunn’s multiple comparison test. All groups, *n* = 6 mice.

**Figure 6:**
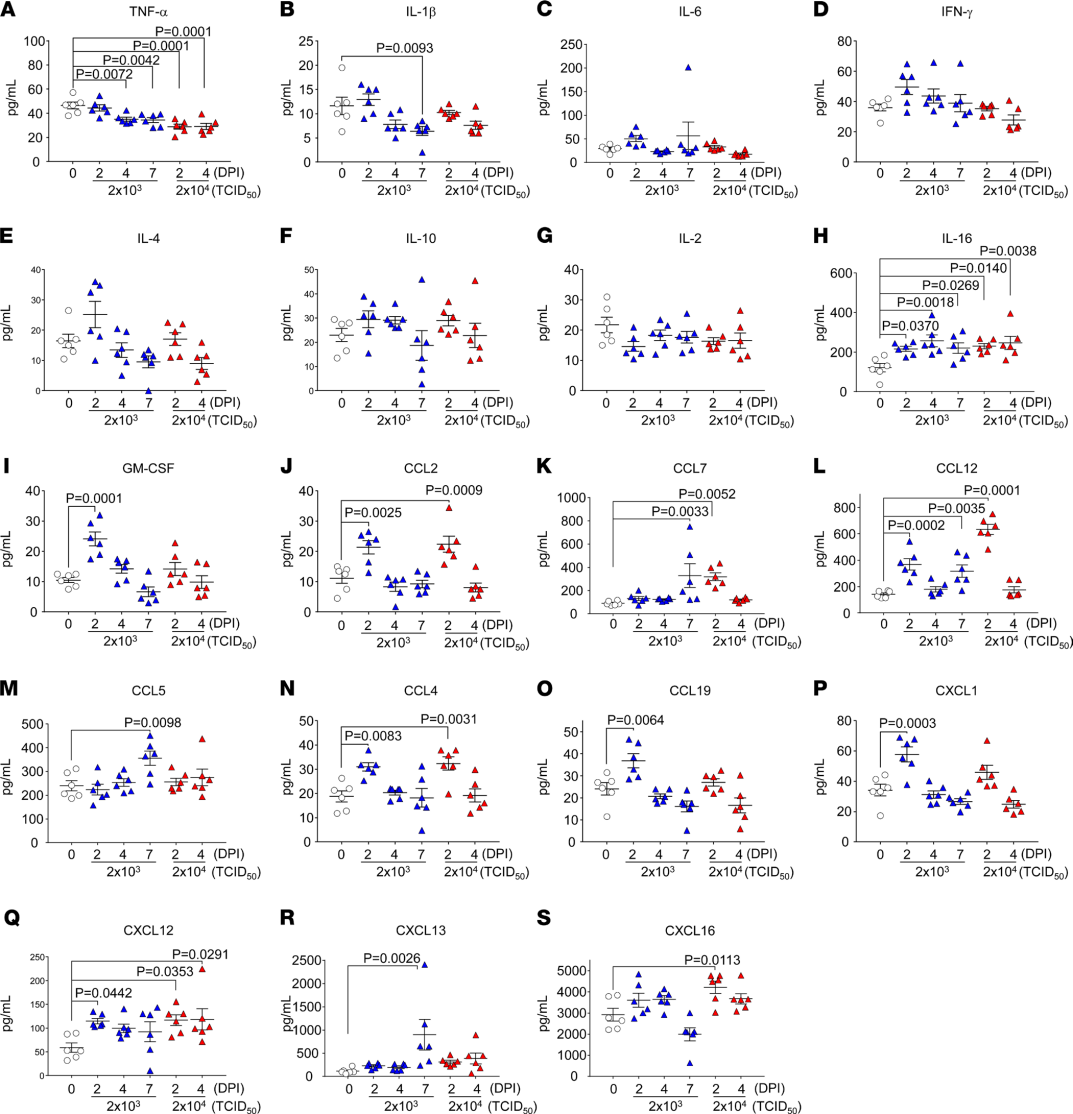
Production of cytokines and chemokines in plasma after SARS-CoV-2 infection. Protein expression of TNF-α (**A**), IL-1β (**B**), IL-6 (**C**), IFN-γ (**D**), IL-4 (**E**), IL-10 (**F**), IL-2 (**G**), IL-16 (**H**), GM-CSF (**I**), CCL12 (**J**), CCL7 (**K**), CCL12 (**L**), CCL5 (**M**), CCL4 (**N**), CCL19 (**O**), CXCL1 (**P**), CXCL12 (**Q**), CXCL13 (**R**) and CXCL16 (**S**) was measured using Bio-Plex assays (Bio-Rad). White triangles indicate the mock infection. Blue and red triangles represent infection doses of 2 × 10^3^ TCID_50_ and 2 × 10^4^ TCID_50_, respectively. Statistical analyses were performed using 1-way ANOVA followed by Dunnett’s multiple comparison test. All groups were *n* = 6 mice.

To demonstrate the inflammatory response in SARS-CoV-2–infected CAG-hACE2 mice, we examined the mRNA levels of various inflammatory cytokines and chemokines in the lung and brain using RT-qPCR and plasma protein levels using multiplex assays. The mRNA expression of various inflammatory cytokines (*Il-6*, *Tnf-*α, *Ifn-*γ*,* and *Ifn-*β) and chemokines (*Ccl2*, *Ccl4*, *Ccl12*, *Cxcl1*, and *Cxcl10*), but not *Il-1*β, was elevated in the lungs at 2 dpi, in the early stage of infection (Figure 4). The elevated cytokines and chemokines in the lungs at 2 dpi were present, along with the peak viral titer (Figure 2, A–C). In contrast, the expression of these mRNAs in the brain was enhanced only at 7 dpi with the elevation of viral RNA (Figure 5). The expression in the blood of IL-16, a pleiotropic cytokine that functions as a chemoattractant and a modulator of T cell activation, was increased at all time points, but not that of other cytokines (TNF-α, IL-1β, IL-6, IFN-γ, IL-4, IL-10, and IL-2) (Figure 6). The chemokines GM-CSF, CCL2, CCL7, CCL12, CCL4, CCL19, CXCL1, and CXCL12 were elevated at 2 dpi in the early stages of infection, and some chemokines were simultaneously increased with mRNA levels. These results indicate that SARS-CoV-2 infection in CAG-hACE2 mice induces severe lung inflammation within a short period and inflammation in the brain at the late stage. To assess the immune cell response in infected CAG-hACE2 mice, FACS analyses were performed using PBMCs (Supplemental Figures 4 and 5) and splenocytes (Supplemental Figures 6 and 7). The results of this analysis showed that CD3^+^, CD4^+^, and CD8^+^ T cell numbers were decreased in infected CAG-hACE2 mice at 7 dpi (middle dose) and at 4 dpi (high dose).

### Characterization of CAG-hACE2 mice.

**Figure 7:**
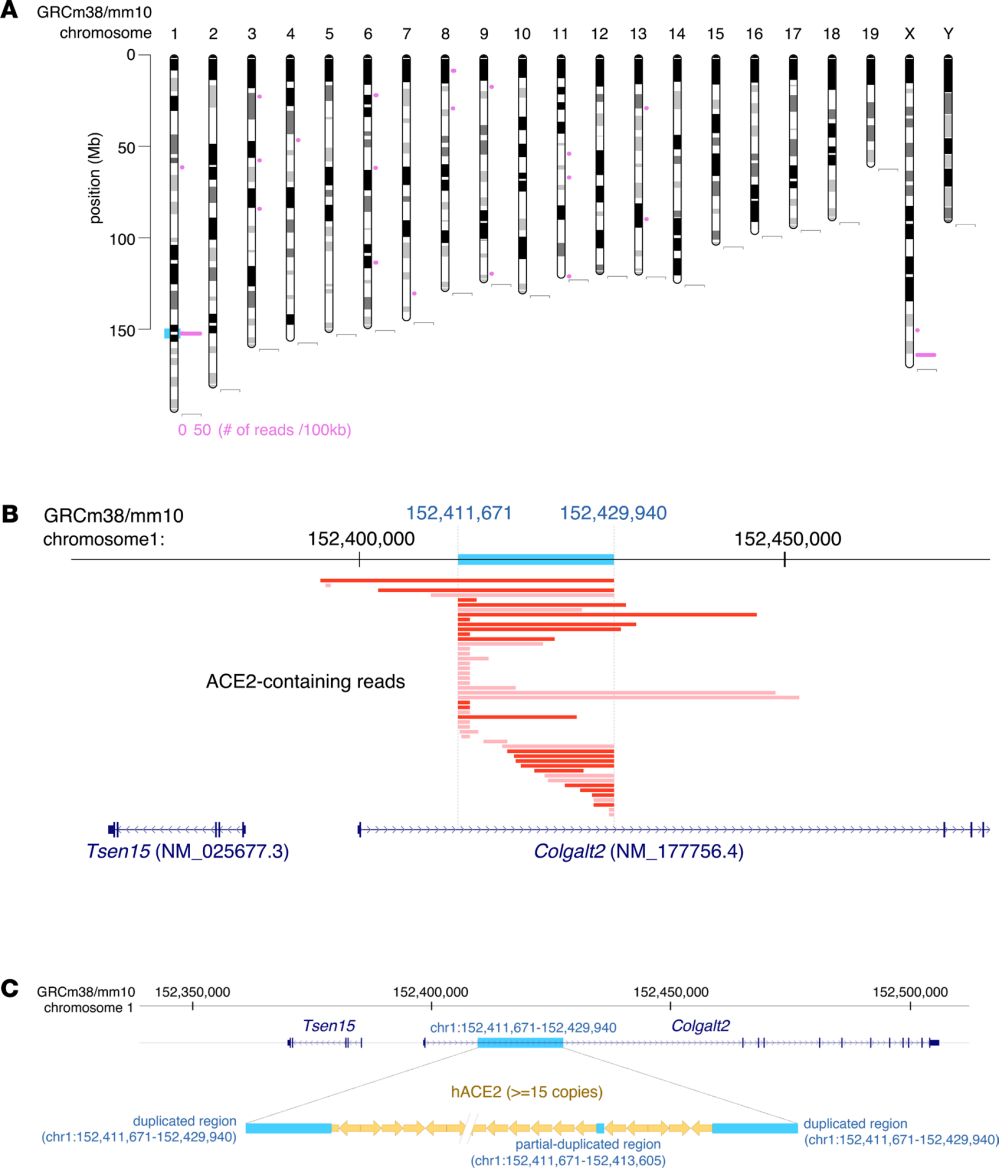
Characterization of CAG-promoter hACE2-transgenic mice. (**A**) Chromosome map of annotated hACE2-containing reads. The pink horizontal bars indicate the number of annotated ACE2-containing reads. Annotated site of integrated hACE2 is indicated as a blue box. (**B**) Detail of annotated site in chromosome 1. The horizontal red and pink bars indicate the ACE2-containing reads mapped to the plus and minus strand of the mouse reference genome (GRCh38/mm10), respectively. The blue box represents the duplicated region. (**C**) Schematic diagrams and sequences are shown as the hACE2-integrated site inside the first intron region of the Colgalt2 gene locus. The blue boxes denote duplicated regions. Each yellow arrow indicates the entire hACE2 sequence. Consensus sequences of reads at 5′- and 3′-junction sites are shown.

To understand the high susceptibility of CAG-hACE2 mice to SARS-CoV-2, we confirmed the integration sites and the copy number of hACE2 genes by continuous long-read sequencing. From the results of the continuous long-read sequence, 131 sequence reads contained hACE2 sequences, and most of these sequence reads were annotated in chromosome 1 and chromosome X (Figure 7A). Some sequence reads were annotated in chromosome X because the intact Ace2 gene was located on chromosome X. In the case of chromosome 1, 49 reads were mapped to a particular site where no ACE2-related genes existed in WT mice (Figure 7B). After detailed confirmation, it was detected that more than 15 hACE2 genes were integrated in tandem into approximately 152,412,000 bp of chromosome 1, which is in the first intron region of Colgalt2 gene locus (Figure 7C). The 152,411,670–152,429,939 bp region of chromosome 1 was duplicated at both sides of the hACE2 integration site. Moreover, a partially duplicated sequence, 152,411,670–152,413,605 bp, was detected in the insertion sequence. These results suggest that multicopy of hACE2 in CAG-hACE2 mice induced high susceptibility to SARS-CoV-2.

### Prevention against SARS-CoV-2 infection by immunization of SARS-CoV-2 RBD protein.

**Figure 8:**
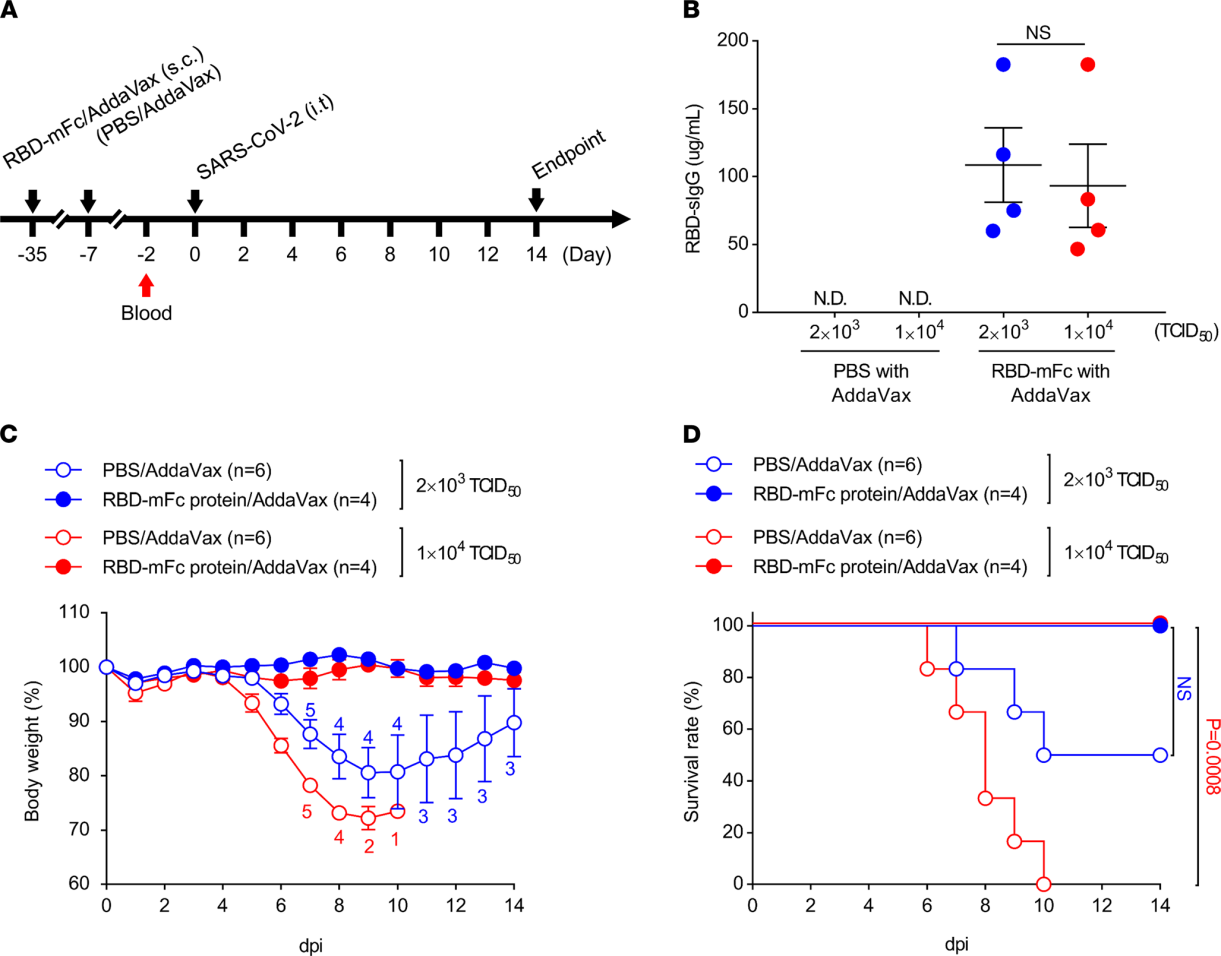
Protection against SARS-CoV-2 by immunization of SARS-CoV-2 RBD protein. (**A**) Schematic showing the experimental schedule. Male and female CAG-hACE2 mice were immunized with RBD-mFc/AddaVax twice before infection and were infected via respiratory tract with SARS-CoV-2 (2 × 10^3^ TCID_50_, *n* = 4; 1 × 10^4^ TCID_50_, *n* = 4). PBS/AddaVax–immunized CAG-hACE2 mice were used as a control (2 × 10^3^ TCID_50_, *n* = 6; 1 × 10^4^ TCID_50_, *n* = 6). (**B**) Expression of RBD-sIgG in plasma from PBS/AddaVax or RBD-mFc/AddaVax–immunized CAG-hACE2 mice at 2 days before infection. Blue and red indicate 2 × 10^3^ TCID_50_ and 1 × 10^4^ TCID_50_, respectively. (**C** and **D**) Percentage of initial body weight (**C**) and survival rate (**D**). Numbers in **C** represent the number of mice measured for body weight at each time. White and colored circles indicate mice injected with PBS/AddaVax or RBD-mFc/AddaVax, respectively. Blue and red denote infection doses of 2 × 10^3^ TCID_50_ and 1 × 10^4^ TCID_50_, respectively. Data are presented as the mean (**B**) and the mean ± SEM (**C**). Statistical analyses were performed using 2-tailed unpaired *t* test for body weight loss (**B** and **C**) and log-rank (mantel-cox) test for survival rate (**D**). *P* < 0.05 for comparison with RBD-mFc/AddaVax (2 × 10^3^ TCID_50_) or PBS/AddaVax–immunized mice, respectively.

It has been reported that immunization with SARS-CoV-2 antigens in mice protects against SARS-CoV-2 infection (22). To confirm the immunization efficacy in CAG-hACE2 and other transgenic mice, the RBDs of SARS-CoV-2 spike protein were subcutaneously administrated with AddaVax in the CAG-hACE2 mice on days –35 and –7, followed by intratracheal infection with SARS-CoV-2 at a dose of 2 × 10^3^ TCID_50_ or 1 × 10^4^ TCID_50_ (Figure 8A). RBD-specific IgG (RBD-sIgG) in the plasma was detected in CAG-hACE2 mice immunized with RBD-Fc/AddaVax (Figure 8B). PBS/AddaVax–treated CAG-hACE2 mice were observed, as was body weight loss from 5 dpi or 6 dpi (2 × 10^3^ TCID_50_ and 1 × 10^4^ TCID_50_, respectively) (Figure 8C). CAG-hACE2 mice with high viral titers gradually died within 10 dpi (Figure 8D). Half of the CAG-hACE2 mice infected with a viral titer of 2 × 10^3^ TCID_50_ also died within 14 days. Importantly, no RBD-mFc/AddaVax–immunized CAG-hACE2 mice infected with viral tiers of 2 × 10^3^ TCID_50_ and 1 × 10^4^ TCID_50_ showed body weight loss or death during this experimental period. These data suggest that the SARS-CoV-2 intratracheally infected CAG-hACE2 mouse model is a useful tool to evaluate vaccine efficacy with regard to SARS-CoV-2.

### Protection against SARS-CoV-2 by intravenous injection of RBD-Fc–immunized mouse plasma.

**Figure 9:**
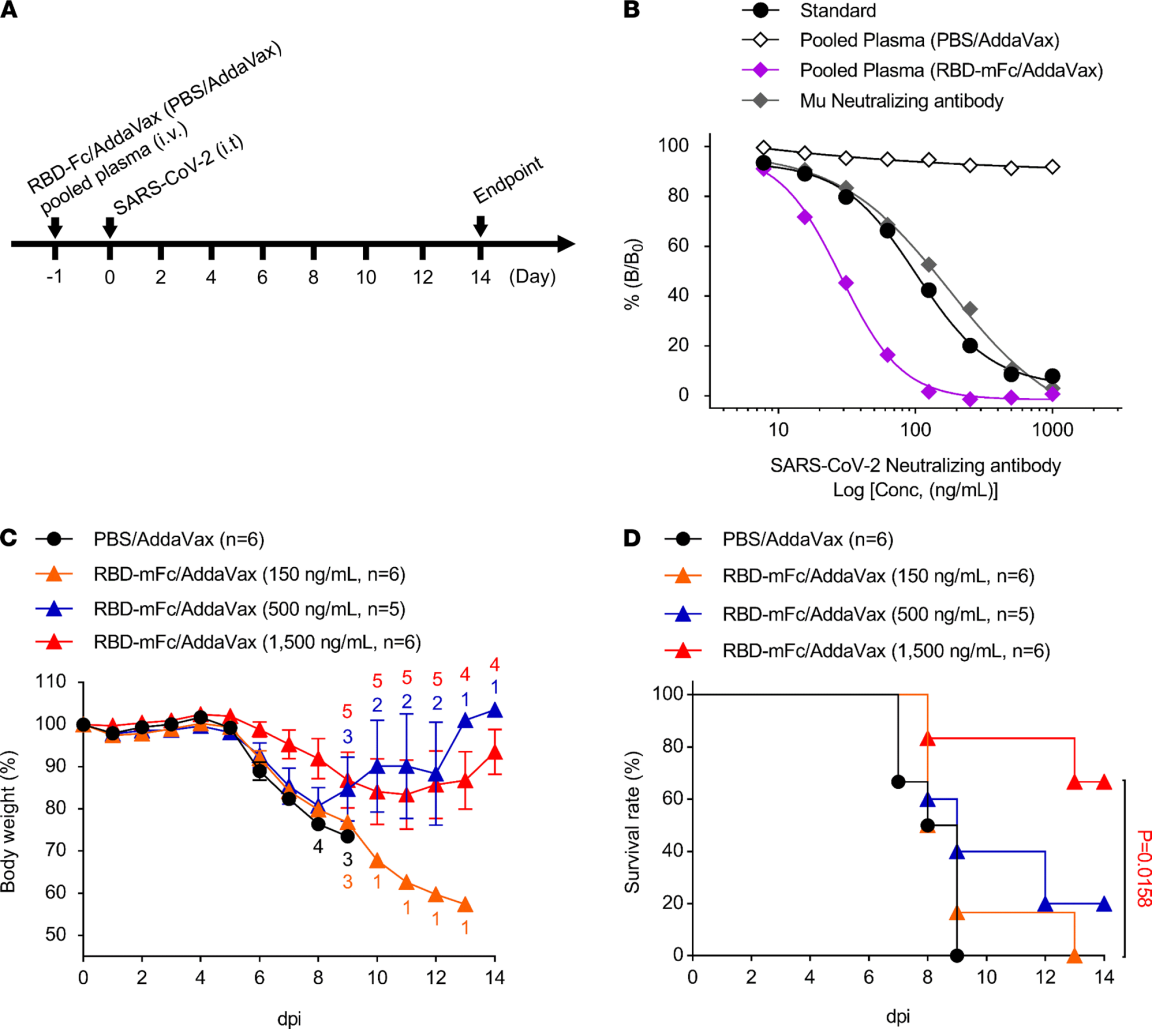
Protection against SARS-CoV-2 infection by the injection of RBD-Fc–immunized mouse plasma. (**A**) Schematic showing the experimental schedule. Male and female CAG-hACE2 mice were intravenously injected with pooled plasma from RBD-mFc/AddaVax–immunized mice at doses of 15 μg/shot (*n* = 6), 50 μg/shot (*n* = 5), and 150 μg/shot (*n* = 6) 1 day before SARS-CoV-2 infection and were infected via respiratory tract with SARS-CoV-2 at a dose of 1 × 10^4^ TCID_50_. CAG-hACE2 mice that were injected with PBS-treated pooled plasma were used as a control (*n* = 6). (**B**) The measurement of SARS-CoV-2–neutralizing antibodies in pooled plasma. Black circles and white, violet, and gray diamonds indicate the standard, pooled plasma from PBS/AddaVax–treated mice, pooled plasma from RBD-mFc/AddaVax–immunized mice, and mouse neutralizing antibody, respectively. (**C** and **D**) Percentage of initial body weight (**C**) and survival rate (**D**). Numbers in **C** represent the number of mice measured for body weight at each time. Black circles and orange, blue, and red triangles indicate plasma from PBS/AddaVax–immunized mice and RBD-mFc/AddaVax–immunized mice at doses of 150 ng/mL (*n* = 6), 500 ng/mL (*n* = 5), and 1500 ng/mL (*n* = 6), respectively. Data were presented as the mean (**B**) and the mean ± SEM (**C**). Statistical analyses were performed using log-rank (mantel-cox) test for survival rate (**D**). *P* < 0.05 for the comparison with PBS-treated pool plasma.

It has been reported that neutralizing antibodies or convalescent plasma from patients with COVID-19 protected against SARS-CoV-2 infection in hACE2-transgenic mice (23, 24). To evaluate the protective effects of plasma from immunized mice, CAG-hACE2 mice were intravenously injected with pooled plasma from RBD-mFc/AddaVax–immunized C57BL/6 mice 1 day before SARS-CoV-2 infection. These mice were infected with SARS-CoV-2 at doses of 2 × 10^3^ TCID_50_ and 1 × 10^4^ TCID_50_ (Figure 9A). SARS-CoV-2–neutralizing antibodies in pooled plasma inhibited the binding of RBD protein to recombinant ACE2 in a concentration-dependent manner (Figure 9B), and the IC_50_ and IC_90_ of RBD-mFc–immunized plasma were 28.51 ng/mL and 88.73 ng/mL, respectively (Table 1). CAG-hACE2 mice injected with PBS/AddaVax–immunized plasma showed body weight loss from 6 dpi, and all of them (*n* = 6) died within 9 dpi (Figure 9, C and D). In contrast, the injection of RBD-mFc/AddaVax–immunized plasma improved body weight loss and death by SARS-CoV-2 infection at a dose-dependent manner. Notably, the injection of RBD-mFc/AddaVax–immunized plasma at a dose of 1500 ng/mL to CAG-hACE2 mice efficiently inhibited body weight loss compared with injection of PBS/AddaVax–immunized plasma at 6–8 dpi. In addition, only 2 of the 6 CAG-hACE2 mice injected with RBD-mFc/AddaVax died during the experimental period, and injection prolonged survival time compared with the mice injected with PBS/AddaVax–immunized plasma. These data also suggest that CAG-hACE2 mice intratracheally infected with SARS-CoV-2 were useful in evaluating therapeutic tools, such as the protective effect of neutralizing antibodies in serum and plasma from convalescent patients with SARS-CoV-2 infection.

## Discussion

Mouse models of SARS-CoV-2 infection with measurable outcomes, such as obvious body weight loss and death, are very useful for evaluating the safety and efficacy of antiviral medicine candidates and vaccine candidates against COVID-19. In this study, a mouse model that is highly susceptible to SARS-CoV-2 using CAG-hACE2 mice was examined. The intratracheal infection with SARS-CoV-2 in CAG-hACE2 mice induced body weight loss, obvious death, and severe acute pneumonia with an increase in proinflammatory cytokines in a viral titer-dependent manner. It was confirmed that at least 15 copies of hACE2 were inserted into the CAG-hACE2 mouse genome. These results indicate that the mouse model used in this study was highly susceptible to SARS-CoV-2. Importantly, immunization with viral antigen or injection of plasma from immunized mice prevented body weight loss and lethality in CAG-hACE2 mice infected with SARS-CoV-2. The data presented here support the idea that CAG-hACE2 mice are beneficial for screening assays for drug and vaccine candidates against SARS-CoV-2 infection.

Mice, which are used as experimental models, are not suitable for an animal model of SARS-CoV-2 infection because SARS-CoV-2 cannot enter into cells using mouse ACE2 receptor (25). The most commonly used SARS-CoV-2–infected mouse models are transgenic mice expressing hACE2, driven by the K18 and HFH4 promoters (14–16, 26). In addition, Hong et al. improved the infection efficiency when evaluating the effectiveness of dexamethasone against SARS-CoV-2 infection in this mouse model (21). Therefore, it is better to evaluate vaccines and medicines using a mouse model that shows clear and constant symptoms of COVID-19. Notably, CAG-hACE2 mice infected with SARS-CoV-2 displayed high morbidity and mortality at infection doses of 2 × 10^2^ to 2 × 10^4^ TCID_50_ (Figure 1, C and D, and Supplemental Figure 1). The viral dose of lethal infection using K18-hACE2 mice was approximately 10^4^ to 10^5^ TCID_50_ or PFU/mouse (17–20). These results suggest that CAG-hACE2 mice are more susceptible to SARS-CoV-2 than other transgenic mice. A comparison of our mouse model with other mouse models that have been reported is presented in Supplemental Table 1. The CAG-hACE2 mice were inserted in tandem at least 15 copies of the hACE2 gene in chromosome1 (Figure 7C). On the other hand, qPCR analysis showed that 8 copies of hACE2 were integrated in the K18-hACE2 mouse genome (27). These results suggest the possibility that the high susceptibility of SARS-CoV-2 in CAG-hACE2 mice is caused by the high expression of hACE2 resulting from multicopy of hACE2 genes. Unfortunately, an accurate number of hACE2 genes integrated in the CAG-hACE2 mouse genome could not be confirmed, despite long-read sequencing.

Immunohistochemical staining results showed high and diffused expression of N protein in the lung at the early stage of infection (2 dpi, Figure 2A). These results are consistent with those of RT-qPCR and viral titers in the lung (Figure 2, B and C). Interestingly, infection with SARS-CoV-2 in the brain was detected at 4 dpi, and viral replication was dramatically increased at 7 dpi, despite intratracheal infection. The upregulation of cytokines and chemokines at the early stage of infection in the lung and at the late stage of infection in the brain were observed (Figures 4 and 5). SARS-CoV-2 infection in the brain was also observed in other hACE2-mice, driven by the K18 and HFH4 promoters (17, 18, 26). Based on these results, SARS-CoV-2 administered via the intratracheal tract initially infected the lungs and was subsequently transmitted to the brain, although the expression of hACE2 in the brain is quite low. It has been reported that SARS-CoV-2 infects the olfactory bulb and neurons (24, 28), where both hACE2 and NRP1 are expressed (29, 30). It has been suggested that SARS-CoV-2 is transmitted from the lung to the brain via the olfactory nervous system through hACE2 and other host factors, such as NRP1. At 7 dpi, despite the viral elimination from the lungs in CAG-hACE2 mice infected with the middle dose (2 × 10^3^ TCID_50_), mortality was approximately 70%. From these results, the lethality of CAG-hACE2 mice infected with a middle dose may be because of brain inflammation caused by an explosive increase in SARS-CoV-2. However, severe injury and elevated cytokine and chemokine levels were observed in the lungs of CAG-hACE2 mice infected with a high dose (2 × 10^4^ TCID_50_), but not in their brains, at 4 dpi (Figures 3–6). The cause of death in CAG-hACE2 mice infected with high doses may be lung injury. However, viral RNA was detected in the brains of deceased CAG-hACE2 mice infected with high-dose SARS-CoV-2 (Supplemental Figure 2B). Therefore, the cause of death in CAG-hACE2 mice infected with high doses is still controversial.

hACE2 was highly expressed in the hearts of CAG-hACE2 mice (Figure 1A). To assess the severity of myocarditis, histopathological analyses were performed on the hearts of CAG-hACE2 mice infected with SARS-CoV-2 (Supplemental Figure 8). The results of these analyses indicated that changes associated with myocarditis were absent. Furthermore, viral RNA was not detected or was very low in the hearts of deceased CAG-hACE2 mice infected with high-dose SARS-CoV-2 (Supplemental Figure 2C). These results suggested that the heart was not the main target of SARS-CoV-2 in CAG-hACE2 mice.

Patients with COVID-19 show some clinical symptoms, such as fever, cough, pneumonia with dyspnea, and bilateral pulmonary infiltration, while some patients develop ARDS and die (31–33). The lungs of CAG-hACE2 mice infected with SARS-CoV-2 at 2 dpi showed mRNA expression of cytokines (*Il-6* and *Tnf-*α), type II interferon (*Ifn-*γ), type I interferon (*Ifn-*β), and chemokines (*Ccl2, Ccl4, Ccl12, Cxcl1* and *Cxcl10*) (Figure 4). The induction of cytokines and chemokines caused by SARS-CoV-2 has also been observed in other mouse models and patients with COVID-19 (17, 34, 35). In particular, cytokines (IL-6 and TNF-α) and chemokines (MCP-1, MIP-1β, KC, and IP-10) are indicators severity of COVID-19 (36, 37). In our mouse model, a local cytokine storm occurred in the lung and brain (Figures 4 and 5), although no systemic cytokine or chemokine production was observed (Figure 6). In addition, immunohistochemical analysis of lungs infected with a high dose of SARS-CoV-2 showed severe pneumonia with progressive infiltration of inflammatory cells and thickness of the alveolar wall (Figure 3 and Supplemental Figure 3). These results seem to be correlated with the expression of inflammatory cytokines and chemokines. These histological findings in CAG-hACE2 mice have also been reported in ARDS lungs of patients with COVID-19 (38, 39). In our system, rapid replication of SARS-CoV-2 in the early stage of infection induces acute inflammation, including elevated cytokines and chemokines, and contributes to lung lesions and lethality, which is in agreement with data in a report describing patients with COVID-19 (40). Furthermore, FACS analyses revealed that the neutrophil count was elevated and lymphopenia-like symptoms were observed in infected CAG-hACE2 mice during the late stage of infection, when the histological scores were high (Supplemental Figures 4–7 and Figure 3B), which was consistent with findings in patients with severe COVID-19 (41). These results suggest that the infection model of CAG-hACE2 mice developed acute severe lung injury accompanied with elevated proinflammatory cytokines and chemokines; therefore, CAG-hACE2 mice provide the possibility for evaluation of medical arms for ARDS with COVID-19.

hACE2 mouse models are considered valuable for evaluating the efficacy of vaccines and medicines. The development of effective vaccines and therapeutic medicines for COVID-19 is still needed worldwide to interrupt the COVID-19 pandemic. The present study has indicated that the vaccination (RBD-mFc, Figure 8) and administration of neutralizing antibodies in the CAG-hACE2 mouse model (pooled plasma from immunized mice, Figure 9) drastically protected against a clear fatality from SARS-CoV-2 infection.

In summary, a potentially novel mouse model of SARS-CoV-2 intratracheal infection can be used to evaluate the preventive and therapeutic effects of vaccines and plasma-containing neutralizing antibodies. The SARS-CoV-2 infection model using CAG-hACE2 mice helps investigation of the protective effect of vaccines and therapeutic medicines, which prevent advances in severity and lethality.

## Methods

Further information can be found in Supplemental Methods.

### Animals.

CAG-hACE2 mice were purchased from the Laboratory Animal Resource Bank of the NIBIOHN (https://animal.nibiohn.go.jp/e_ace2_tg_17.html). C57BL/6 mice were purchased from CREA Japan Inc. To maintain the heterozygous (hACE2Tg/+) hACE2 mice, WT and heterozygous (hACE2Tg/+) hACE2 mice were mated. The genotypes of mice were determined by PCR for ear DNA using the primer sets 5′-CTTGGTGATATGTGGGGTAGA-3′ and 5′-CGCTTCATCTCCCACCACTT-3′. Male and female CAG-hACE2 mice and C57BL6 mice (weighing 18–24 g) were maintained in plastic cages with free access to food and water and housed at 25°C ± 2°C with a 12-hour-light/dark cycle. All experiments with live SARS-CoV-2 were performed in enhanced animal biosafety level 3 containment laboratories at the Tsukuba Primate Research Center of the NIBIOHN.

### Virus.

VeroE6/TMPRSS2 was obtained from the JCRB Cell Bank of NIBIOHN (https://cellbank.nibiohn.go.jp/~cellbank/cgi-bin/search_res_det.cgi?RNO=JCRB1819). SARS-CoV-2 (Tokyo strain, UT-NCGM02) was provided in-house (National Center for Global Health and Medicine). To propagate SARS-CoV-2, the virus was infected with VeroE6/TMPRSS2 at an MOI of 0.01 and then cultured in DMEM containing 2% FBS at 37°C for 3 days. The culture media were stored at –80°C and used for experimental infection in CAG-hACE2 mice at the NIBIOHN. All experiments with live SARS-CoV-2 were performed in enhanced BSL3 containment laboratories at the Tsukuba Primate Research Center of the NIBIOHN, which followed the approved standard operating procedures of the BSL3 facility.

### Western blotting.

Organs of C57BL/6 mice and CAG-hACE2 mice were homogenized in 1 mL RIPA buffer using MagNA Lyser and Green Beads (Roche) at 6500 rpm for 25 seconds 4 times. Lysates were centrifuged at 21,800*g* for 5 minutes at room temperature. The supernatant of the lysate was denatured with sample buffer solution with reducing reagent (6×) for SDS-PAGE (Nacalai Tesque). Proteins (40 μg protein per well) were subjected to SDS-PAGE electrophoresis using 4 %–20% Mini-PROTEAN TGX Precast Protein Gel (Bio-Rad) and transferred to a polyvinylidene difluoride membrane (Immobilon-P Membrane, Merck). The membranes were blocked with 5% skim milk for 1 hour at room temperature and then incubated with anti-human ACE2 antibody (1:1000, catalog MAB9331, clone 171608, R&D Systems) or anti-GAPDH polyclonal antibody (1:1500, catalog ab9485, Abcam Cambridge) at 4°C overnight. Then, the membranes were incubated with HRP-conjugated goat anti-mouse polyclonal IgG for hACE2 (1:2000, catalog 172-1011, Bio-Rad) or HRP-conjugated goat anti-rabbit polyclonal IgG for GAPDH (1:2000, catalog 172-1019, Bio-Rad) for 1 hour at room temperature, and the protein signals were detected using a chemiluminescence detection reagent (Chemi-Lumi One L; Nacalai Tesque Inc.) and visualized using the ImageQuant LAS 4000 system (Cytiva).

### Identification of the hACE2 gene integration site.

The genome of CAG-hACE2 mice was purified from the liver using the Wizard HMW DNA Extraction Kit (Promega). Purified DNA was analyzed using a continuous long read of PacBio Sequel II (Promega) (accession DRR315201). First, whole data of subreads were extracted from continuous long reads using the manufacturer’s tool, bam2fastq. Next, using minimap2 2.27 (42), we mapped all extracted subreads to the sequence of the SalI-StuI fragment from pCAGGS-P7-hACE2 as an integrated hACE2 reference sequence with a CAG enhancer/promoter and poly A signal region. After this step, only these ACE2-containing subreads were mapped to the mouse genome GRCh38/mm10 by minimap2. Some reads were mapped to the intact mouse ACE2 region in the X chromosome, while the others were mapped to integrated sites without ACE2 genes in the WT mouse genome GRCh38/mm10. Detailed structures of the integrated sites were confirmed and reconstructed using Blat v.36 (43), and the results of Blat were illustrated using R 3.5.1 (https://www.r-project.org).

### SARS-CoV-2-infected CAG-hACE2 mice via the respiratory tract.

Mice were assigned randomly to 2 groups in WT mice and 4 groups in CAG-hACE2 mice to assess infection (PBS treatment) and infection: WT + PBS (*n* = 3), WT + 2 × 10^4^ TCID_50_ (*n* = 4), hACE2-transgenic + PBS (*n* = 3), hACE2-transgenic + 2 × 10^2^ TCID_50_ (*n* = 5), hACE2-transgenic + 2 × 10^3^ TCID_50_ (*n* = 6), and hACE2-transgenic + 2 × 10^4^ TCID_50_ (*n* = 5). Following intubation with a bronchial tube that was inserted into WT and CAG-hACE2 mice under anesthesia by an otoscope (100 μL/mouse, medetomidine, 20 μg/mL; midazolam, 600 μg/mL; butorphanol, 1 mg/mL), WT and CAG-hACE2 mice were infected via respiratory tract with SARS-CoV-2 stock virus at dosages of 2 × 10^2^ TCID_50_/25 μL, 2 × 10^3^ TCID_50_/25 μL, and 2 × 10^4^ TCID_50_/25 μL using a 100 μL micropipette. Mock infection control mice were administrated via respiratory tract with an equal volume of PBS. Infected mice were intraperitoneally injected with atipamezole (100 μL/mouse, 20 μg/mL). Infected mice were recorded daily for body weight and survival. Mice that were clearly emaciated were euthanized after recording their body weight. CAG-hACE2 mice were sacrificed at 0, 2, 4, and 7 dpi to evaluate viral replication, mRNA expression of inflammatory cytokines, and histopathological changes.

### Measurement of SARS-CoV-2 replication by TCID_50_.

To measure the viral titer in the lung and brain, organs were homogenized in 3 mL RPMI1640 medium containing penicillin-streptomycin using gentleMACS Dissociator and M tubes (Miltenyi Biotec). Organ lysates were divided into 250 μL for RNA purification and 2 mL for TCID_50_ of SARS-CoV-2. Organ lysates were serially diluted by a factor of 10 with RPMI1640 containing 5% FBS and penicillin-streptomycin. The diluted lysates were incubated with VeroE6/TMRPSS2 cells (2 × 10^4^ cells/well) in 96-well plates for 3 days, and viral titers of each organ sample were calculated using the Reed-Muench calculation method.

### Purification of RNA and RT-PCR.

Organ RNA was purified using TRIzol LS Reagent (Thermo Fisher Scientific) according to the manufacturer’s protocol. Reverse transcriptase (RT) reactions were conducted with ReverTra Ace qPCR RT master mix with gDNA remover (TOYOBO) using 500 ng organ RNA. To quantify the SRAS-CoV-2 replication, the RT reaction products were diluted, and 1 of 5 and 2 μL of the diluents were subjected to quantitative real-time PCR using THUNDERBIRD Probe qPCR Mix (TOYOBO). The primer sets and probe sequences were as follows: 5′-AAATTTTGGGGACCAGGAAC-3′ (forward primer), 5′-TGGCAGCTGTGTAGGTCAAC-3′ (reverse primer), and 5′-FAM-ATGTCGCGCATTGGCATGGA-BHQ-3′ (probe). To quantify the expression of cytokine and chemokine genes, real-time PCR was conducted using THUNDERBIRD Next SYBR qPCR Mix (TOYOBO). The expression levels of each gene and viral RNA were quantified using a QuantStudio 3 real-time PCR system (Thermo Fisher Scientific). Primer sets used to detect cytokine and chemokine genes are shown in Supplemental Table 2.

### Multiplex cytokine and chemokine assays.

Cytokine, proinflammatory, and chemokine proteins in plasma were measured using a Bio-Plex Pro (Mouse Chemokine 31-plex panel, Bio-Rad), following the manufacturer’s instructions. A multiplex assay was performed in BSL-3, and acquired data were analyzed using the Bio-Plex 200 system.

### Purification of the RBD-Fc proteins.

A DNA sequence encoding the RBD1 region (319–541 amino acids) of the spike protein of SARS-CoV-2 (accession QHD43416.1) was designed by codon optimization, synthesized, and subcloned into a pcDNA3.1-based expression vector with the Fc region of mouse IgG2a or human IgG1 at the C-terminus. For RBD-Fc expression, the plasmid was transfected into Expi293F cells using the Expi293 Expression System (Thermo Fisher Scientific), and the cell culture supernatants were harvested. The produced RBD-Fc fusion protein was purified by affinity chromatography using a Protein A HP column (Cytiva) (44).

### Protection against SARS-CoV-2 by immunization with SARS-CoV-2 RBD protein.

RBD-mFc (10 μg/mouse) or PBS was mixed with AddaVax (Th1/Th2 adjuvant, Invitrogen), and these mixtures were subcutaneously injected into CAG-hACE2 mice on days –35 and –7 (Figure 8A). Blood samples were taken with heparin sodium 2 days before SARS-CoV-2 infection to evaluate the production of RBD-sIgG. Mice were randomly assigned to 2 groups, PBS/AddaVax–immunized mice and RBD-mFc protein/AddaVax–immunized mice before SARS-CoV-2 infection: PBS/AddaVax with 2 × 10^3^ TCID_50_ (*n* = 6), RBD-mFc protein/AddaVax with 2 × 10^3^ TCID_50_ (*n* = 4), PBS/AddaVax with 2 × 10^4^ TCID_50_ (*n* = 6), and RBD-mFc protein/AddaVax with 2 × 10^3^ TCID_50_ (*n* = 4), respectively. RBD-immunized CAG-hACE2 mice and PBS-treated CAG-hACE2 mice were infected with 2 × 10^3^ TCID_50_/25 μL and 1 × 10^4^ TCID_50_/25 μL SARS-CoV-2 using the same method as above. The body weight and survival of the infected mice were monitored for up to 14 days.

### Detection of RBD-sIgG.

The 96-well flat-bottom plates were coated with 100 μg/well SARS-CoV-2 RBD-hFc protein overnight at 4°C. Plates were washed 5 times with PBS containing 0.05% Tween 20 and blocked for 2 hours at room temperature with 3% BSA/PBS. After blocking, the SARS-CoV-2 spike–neutralizing antibody (catalog 40592-MM57, clone 57, SinoBiological) was diluted 2-fold from the top concentration to 7 points as a standard, and the diluted standard and each plasma sample (100 μL) were incubated for 1 hour at room temperature. The specific IgG was detected by incubation with HRP-conjugated goat anti-mouse IgG (1:30,000) (catalog 172-1011, Bio-Rad) for 1 hour, followed by incubation with peroxidase substrate (TMB, SeraCare Life Sciences). The enzymatic reactions were stopped using sulfuric acid (TMB Stop Solution, SeraCare Life Sciences). Optical density values were measured at 450 nm using a microplate reader (Multiskan FC, Thermo Fisher Scientific).

### Detection of SARS-CoV-2–neutralizing antibodies in plasma.

To prepare RBD-immunized pooled plasma, RBD-mFc (10 μg/mouse) or PBS was mixed with AddaVax, and these mixtures were subcutaneously injected into C57BL/6 mice on days 0 and 28. Plasma was collected from RBD-mFc–immunized mice on day 35 under anesthesia. Detection of SARS-CoV-2–neutralizing antibody was performed with the SARS-CoV-2 Neutralizing Antibody Detection ELISA Kit (Cayman) and calculated as half maximal inhibition concentration (IC_50_) and IC_90_. The SARS-CoV-2 spike–neutralizing antibody was also used to validate the effectiveness of this kit against mouse antibodies. The administration volume of RBD-immunized plasma was determined by IC_90_ and estimated from the circulating blood volume of the mice.

### Protection against SARS-CoV-2 by transfer of RBD-Fc–immunized mouse plasma.

CAG-hACE2 mice were assigned randomly to 4 groups to assess the protective efficacy of neutralizing antibodies against SARS-CoV-2 infection: pooled plasma (PBS/AddaVax, *n* = 6), RBD-mFc/AddaVax 150 ng/mL (*n* = 6), RBD-mFc/AddaVax 500 ng/mL (*n* = 5), and RBD-mFc/AddaVax 1500 ng/mL (*n* = 6). RBD-immunized pool plasma (100 μL/shot) were intravenously injected into CAG-hACE2 mice 1 day before SARS-CoV-2 infection. PBS-treated plasma was used as the negative control. Mice were infected with 1 × 10^4^ TCID_50_/25 μL SARS-CoV-2 using the same method as above. The body weight and survival rates of the infected mice were monitored for up to 14 days.

### Macroscopic and histological evaluations.

Brain and lung samples were collected at 0 (control), 2, 4, and 7 dpi. These organs were immersed in 10% formalin for 24 hours, embedded in paraffin, and cut into 2 and 4 μm (brain and lung, respectively) sections onto a slide glass (Matsunami Glass). Tissue sections were stained with H&E and observed using a BZ-9000 microscope (HS All-in-One Fluorescence Microscope, Keyence) at magnifications of ×200, ×400, and ×1000. Images were created using multistack module tool software at a magnification of ×100. Three randomly chosen fields of each section were graded under a BZ-9000 microscope at a magnification of ×200 for semiquantitative assessment of lung injury using a previously defined score, consisting of alveolar congestion, hemorrhage, neutrophil infiltration, alveolar wall thickness, and hyaline membrane formation. Grade details are as follows: 0, minimal (little) damage; 1, mild damage; 2, moderate damage; 3, severe damage; and 4, maximal damage (45). The average score was used for the individual lung injury score, and all evaluations were performed by 2 investigators who were blinded to the experimental groups.

### Immunohistochemical analyses.

Brain and lung samples were collected at 0 (control), 2, 4, and 7 dpi. These organs were immersed in 10% neutralized formalin for 24 hours, embedded in paraffin, and cut into 2 and 4 μm (brain and lung, respectively) sections onto Superfrost Plus slides (Matsunami Glass). Immunohistochemical procedures were performed as follows: Tissue sections were subjected to antigen retrieval with HistoVT One (Nacalai Tesque) at 90°C in a warm bath for 30 minutes, and endogenous peroxidase was inactivated with 0.3% H_2_O_2_/methanol for 30 minutes after rinsing with TBS. The slide-mounted sections were treated with 5% normal donkey serum/PBS (Jackson ImmunoResearch Laboratories) for 2 hours at room temperature, followed by incubation with avidin and biotin solution (Vector Laboratories) for 15 minutes at room temperature. The slide-mounted sections were then incubated with a rabbit anti-SARS-CoV-2 nucleocapsid monoclonal antibody (catalog GTX635679, clone HL344, GeneTex) for 30 hours at room temperature, followed by 2 rinses with TBS containing 0.1% Tween20 and 1 with TBS. To visualize the target protein expression, the sections were incubated with the appropriate secondary antibody (catalog 711-065-152, Biotin-SP-AffiniPure Donkey Anti-Rabbit IgG, Jackson ImmunoResearch Laboratories) for 1 hour at room temperature. The sections were then incubated with peroxidase streptavidin (Jackson ImmunoResearch Laboratories) for 20 minutes at room temperature, followed by ImmPACT (DAB Peroxidase Substrate Kit, Vector Laboratories) for 5 minutes at room temperature. The slides were counterstained with Mayer’s hematoxylin solution (Nacalai Tesque). No specific immunostaining was observed in any of the control sections. Immunostaining was observed using a BZ-9000 microscope (Keyence) at a magnification of ×400. Images were created using multistack module tool software at magnifications of ×40 and ×100 (brain and lung, respectively).

### Statistics.

Data are presented as the mean ± SEM. Statistical analyses were performed using GraphPad Prism 7.0. One-way ANOVA followed by Dunnett’s multiple comparison test was used to determine the viral titer per organ (TCID_50_) and the production of cytokines and chemokines. Kruskal-Wallis 1-way ANOVA followed by Dunn’s multiple comparison test was used for the viral mRNA copies per organ, histological score, and the mRNA expression of cytokines and chemokines. The unpaired 2-tailed Student’s *t* test was used for RBD-sIgG. The log-rank test was used to determine the survival rate. Statistical significance was set at *P* < 0.05.

### Study approval.

All animal studies were approved by the Animal Care and Use Committee of the NIBIOHN.

## Author contribution

MNA, DU, and YY conceived and designed the study. MNA, DU, KK, and TY performed the experiments. YN analyzed the long-read sequencing data. HK and SN provided the purified proteins. YK provided the viruses essential for this study. MNA, DU, and YY wrote and edited the manuscript. YY supervised the study. All authors read and approved the final manuscript. Co–first authors MNA and DU contributed equally to all experiments in this study. MNA is listed first in the authorship list because of MNA’s greater contribution to acquiring grant funding.

## Supplementary Material

Supplemental data

## Figures and Tables

**Table 1:**
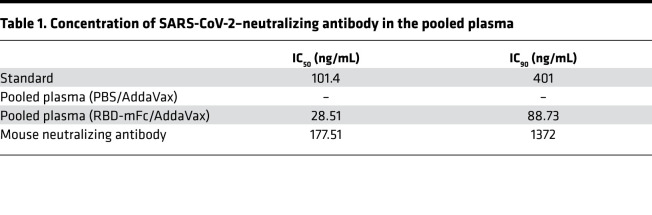
Concentration of SARS-CoV-2–neutralizing antibody in the pooled plasma

## Version 1 08/31/2021

In-Press Preview

## Version 2 10/08/2021

Electronic publication
