# Supplementary material for: Highly susceptible SARS-CoV-2 model in CAG promoter–driven hACE2-transgenic mice
Source: JCI Insight. 2021 Oct 8;6(19):e152529. doi: 10.1172/jci.insight.152529 (PMC8525594; doi:10.1172/jci.insight.152529)
Supplement: Supplemental data [file jciinsight-6-152529-s214.pdf]

Supplemental Material

Highly susceptible SARS-CoV-2 model in CAG promoter-driven hACE2 transgenic mice

Masamitsu N Asaka[1#], Daichi Utsumi[1#], Haruhiko Kamada[2,3,4], Satoshi Nagata[3], Yutaka Nakachi[5], Tomokazu Yamaguchi[6], Yoshihiro Kawaoka[7,8,9], Keiji Kuba[6], Yasuhiro Yasutomi[1]*

[1] Laboratory of Immunoregulation and Vaccine Research, Tsukuba Primate Research Center, National Institutes of Biomedical Innovation, Health and Nutrition, 305-0843 Tsukuba, Ibaraki, Japan
[2] Laboratory of Biopharmaceutical Research, National Institutes of Biomedical Innovation, Health and Nutrition, Ibaraki, Osaka, Japan
[3] Laboratory of Antibody Design, National Institutes of Biomedical Innovation, Health and Nutrition, Ibaraki, Osaka, Japan
[4] Global Center for Medical Engineering and Informatics, Osaka University, Suita, Osaka, Japan
[5] Department of Molecular Brain Science, Graduate School of Medical Sciences, Kumamoto University, Kumamoto, Japan
[6] Department of Biochemistry and Metabolic Science, Graduate School of Medicine, Akita University, Akita, Japan
[7] Influenza Research Institute, Department of Pathobiological Sciences, School of Veterinary Medicine, University of Wisconsin, Madison, USA
[8] Institute of Medical Science, University of Tokyo, Tokyo, Japan
[9] National Center for Global Health and Medicine, Tokyo, Japan
[#]These authors contributed equally to this work.

*Corresponding author:
Yasuhiro Yasutomi, DVM., Ph.D.
Laboratory of Immunoregulation and Vaccine research, Tsukuba Primate Research Center,

National Institutes of Biomedical Innovation, Health and Nutrition (NIBIOHN) 1-1 Hachimandai, Tsukuba, Ibaraki, 305-0843, JAPAN
Tel: +81-29-837-2073
E-mail: yasutomi@nibiohn.go.jp

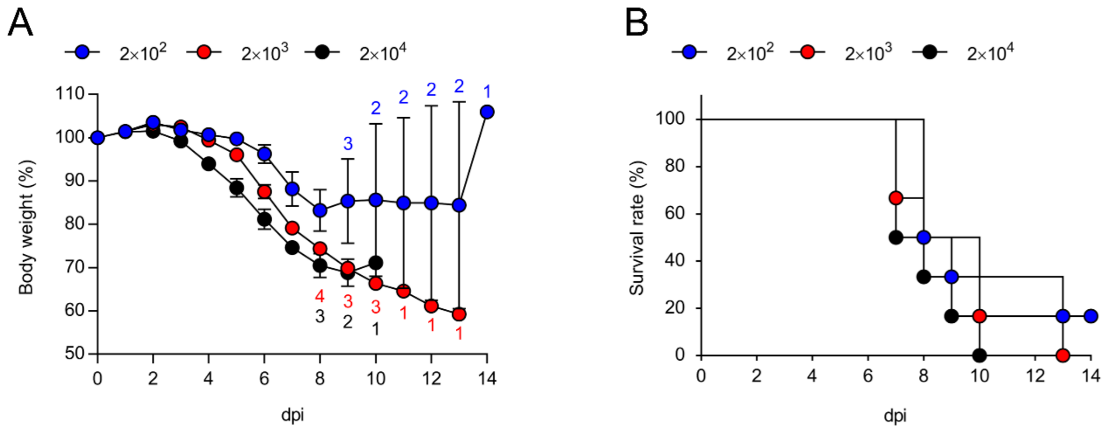

Supplemental Figure 1. Intranasal infection of SARS-CoV-2 with CAG-hACE2 mice.

A and B, CAG-hACE2 mice were intranasally infected with SARS-CoV-2 (2×10^2 $TCID_{50}$: n=6, 2×10^3 $TCID_{50}$: n=6 and 2×10^4 $TCID_{50}$: n=6). Percentage of initial body weight (A), and survival rate (B) were monitored for up to 14 days. Blue, red, and black circles indicate 2×10^2 $TCID_{50}$, 2×10^3 $TCID_{50}$, and 2×10^4 $TCID_{50}$, respectively.

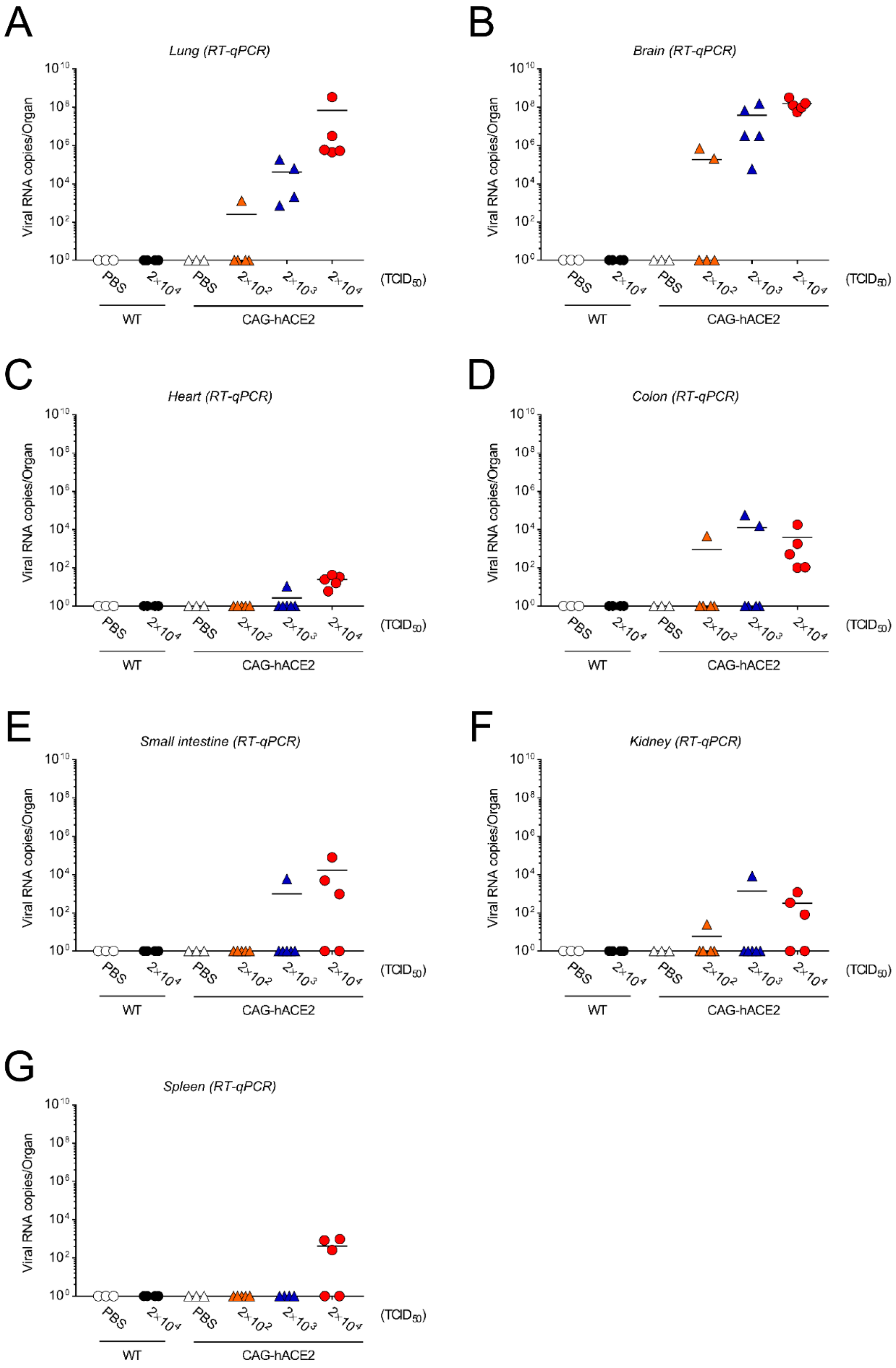
A
Lung (RT-qPCR)
B
Brain (RT-qPCR)
C
Heart (RT-qPCR)
D
Colon (RT-qPCR)
E
Small intestine (RT-qPCR)
F
Kidney (RT-qPCR)
G
Spleen (RT-qPCR)
Viral RNA copies/Organ
10^{0}
10^{2}
10^{4}
10^{6}
10^{8}
10^{10}
PBS
2x10⁴
2x10²
2x10³
(TCID50)
WT
CAG-hACE2

Supplemental Figure 2. Viral copy levels in organs due to SARS-CoV-2 intratracheally infection. qRT-PCR of SARS-CoV-2 N gene expression in lung (A), brain (B), heart (C), colon (D), small intestine (E), kidney (F) and spleen (G). These samples were collected at 6 dpi (n=1) and 7 dpi (n=4) in high viral dose, at 7 dpi (n=1), 8 dpi (n=1), 11 dpi (n=2) and 14 dpi (n=2) in middle viral dose, and at 12 dpi (n=1) and 14 dpi (n=4) in low viral dose, respectively. Orange triangles, blue triangles and red circles are indicated as 2×10^2 $TCID_{50}$, 2×10^3 $TCID_{50}$ and 2×10^4 $TCID_{50}$, respectively. Data are presented as the mean ± SEM.

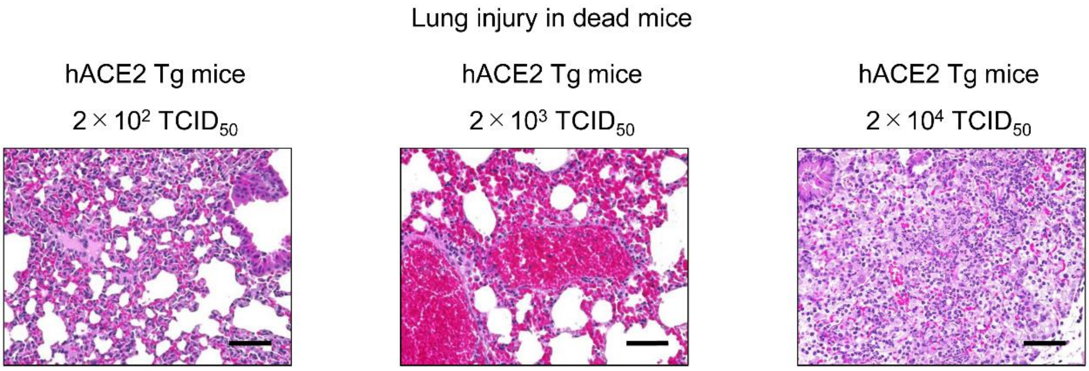

Supplemental Figure 3. Severe lung injury in hACE2 Tg mice caused by SARS-CoV-2 infection. H&E staining of representative images are shown in the lung tissues of deceased mice infected with 2×10^2 $TCID_{50}$ (left panel), 2×10^3 $TCID_{50}$ (center panel) and 2×10^4 $TCID_{50}$ (right panel) SARS-CoV-2. Bars indicates 50 μm.

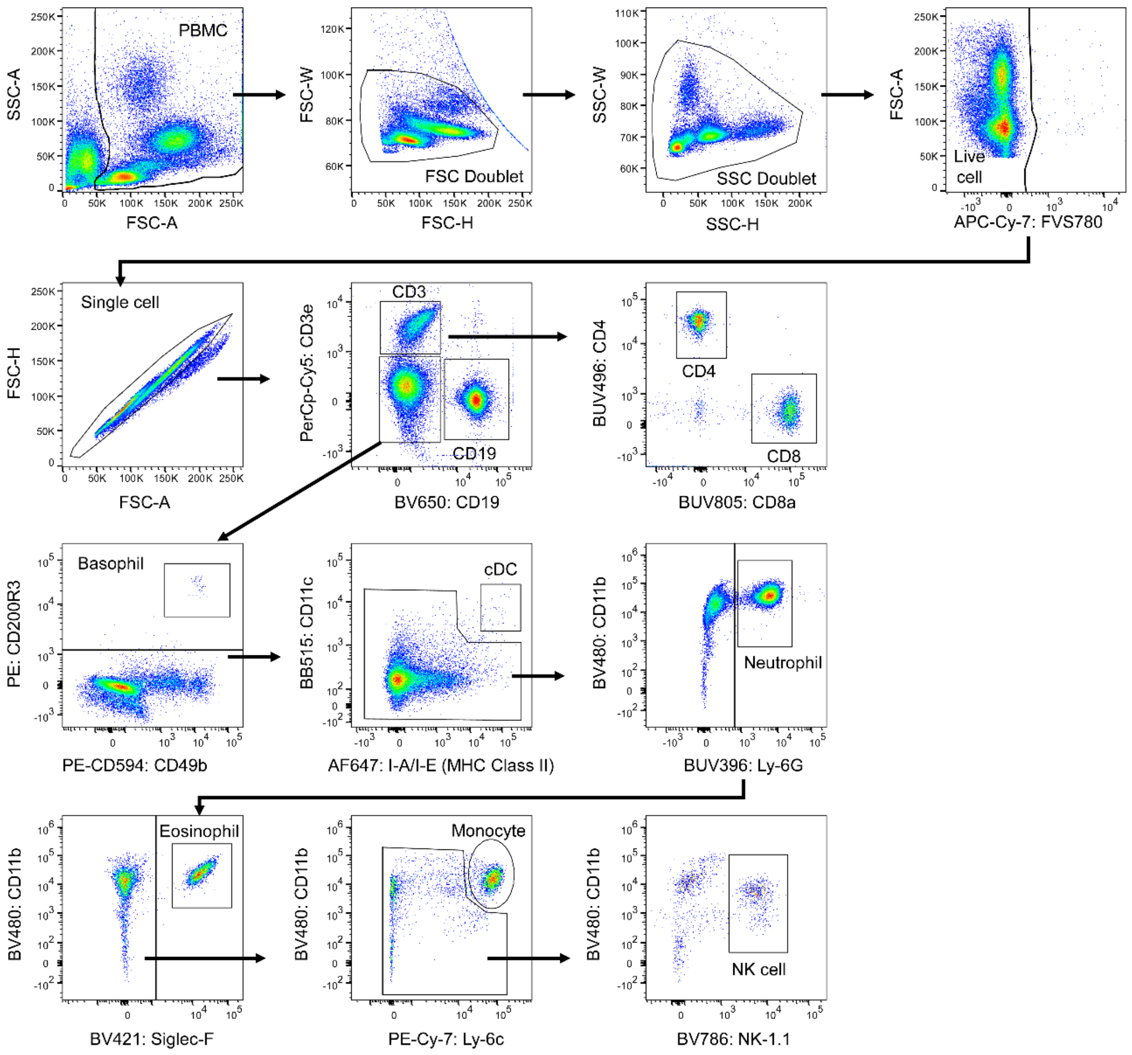

Supplemental Figure 4. Gating of FACS analyses of PBMC. Gating strategy relative to the quantification of the immune cells in PBMC of CAG-hACE2 Tg mice with SARS-CoV-2 infection. The analysis was performed by acquiring a single cell of 50,000 events. Figure was showed the representative sample. The population of immune cells are as shown follows; CD3 + T cell, CD4+ T cell, CD8+ T cell, CD19+ B cell, Basophil (CD200R3+, CD49b+), conventional dendritic cell (cDC: CD11c+, I-A/I-E+), Neutrophil (CD11b+, Ly-6G), Eosinophil (CD11b+, Siglec-F+), Monocyte (CD11b+, Ly-6c) and NK

cell (CD11b+, NK-1-1+).

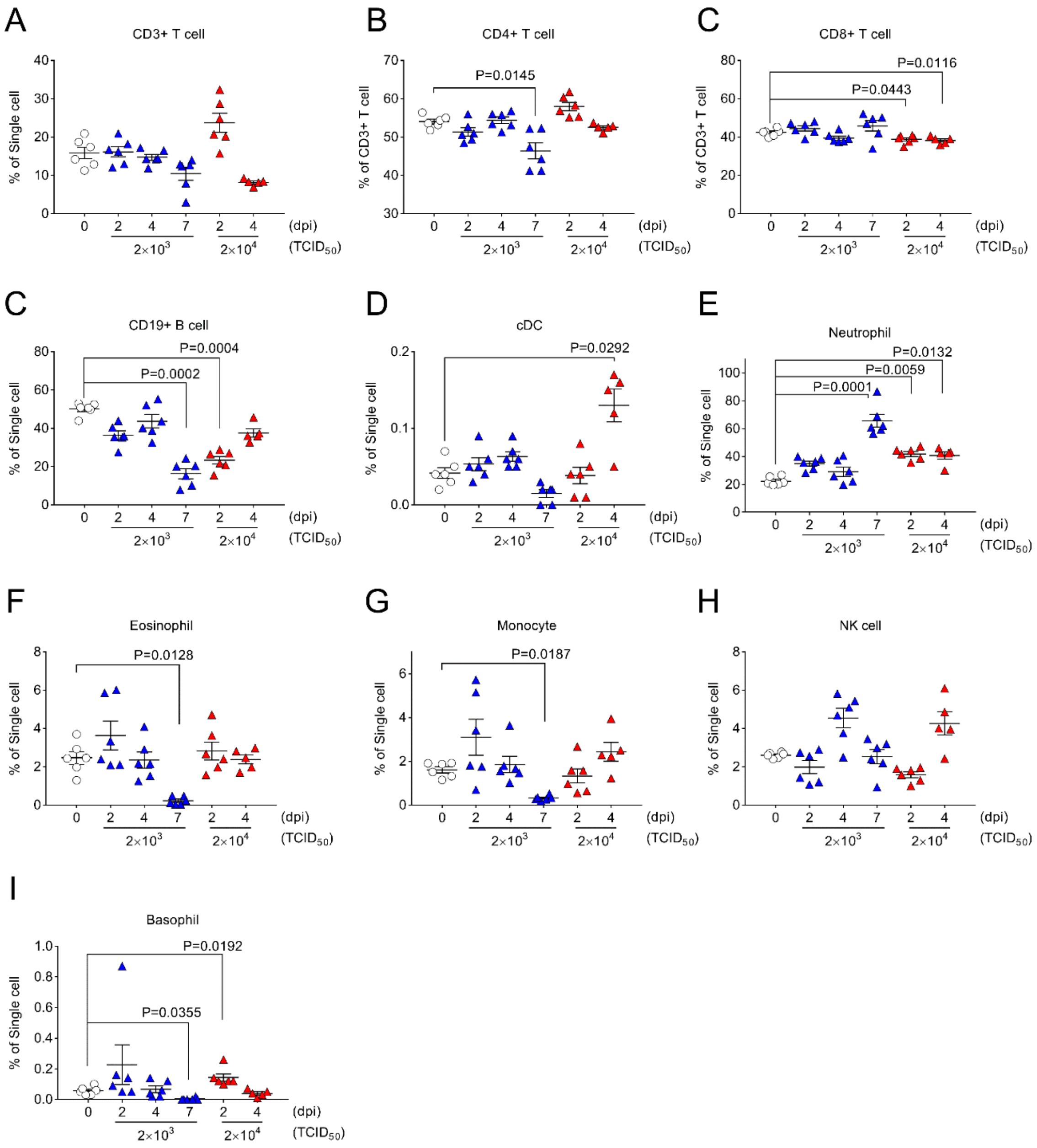

Supplemental Figure 5. Results of FACS analyses using PBMC in infected CAG-hACE2 mice. A-I, The population of immune cells are as shown in Sup Fig. 4. White triangles indicate the mock infection (0 dpi, n=6). Blue and red triangles indicate the

infection dose of 2×10³ TCID$_{50}$ (2, 4 and 7 dpi, n=6) and 2×10⁴ TCID$_{50}$ (2 dpi, n=6. 4 dpi, n=5), respectively. Data are presented as the mean ± SEM. Statistical analyses were performed using Kruskal-Wallis one-way ANOVA followed by Dunn's multiple comparison test.

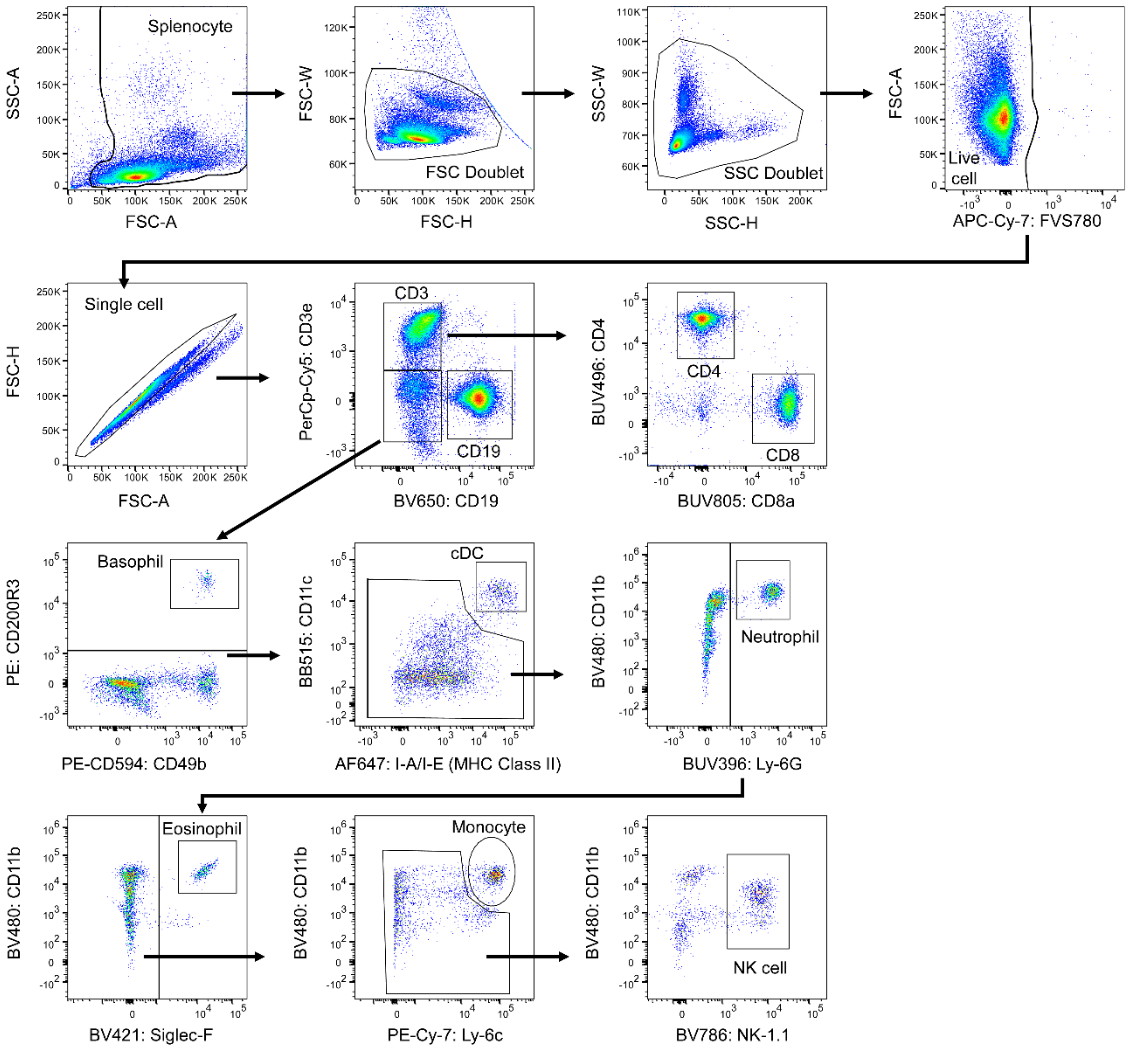

Supplemental Figure 6. Gating of FACS analyses of splenocytes. Gating strategy relative to the quantification of the immune cells in PBMC of CAG-hACE2 Tg mice with SARS-CoV-2 infection. The analysis was performed by acquiring a single cell of 50,000

events. Figure was showed the representative sample.

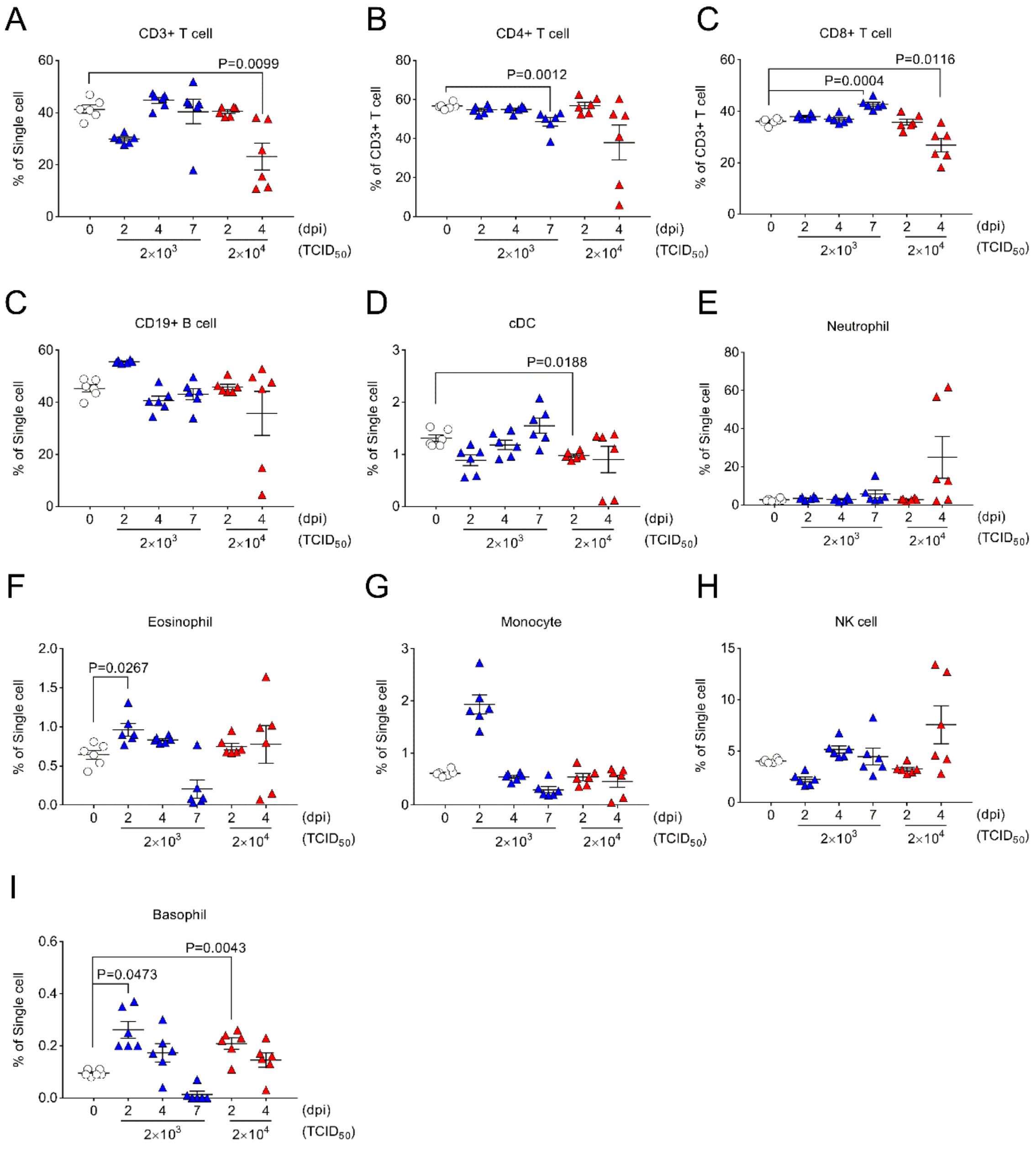

Supplemental Figure 7. Results of FACS analyses using splenocytes in infected CAG-hACE2 mice. A-I, The population of immune cells are as shown in Sup Fig. 5; CD3 + T cell, CD4+ T cell, CD8+ T cell, CD19+ B cell, Basophil (CD200R3+, CD49b+),

conventional dendritic cell (cDC CD11c+, I-A/I-E+), Neutrophil (CD11b+, Ly-6G), Eosinophil (CD11b+, Siglec-F+), Monocyte (CD11b+, Ly-6c) and NK cell (CD11b+, NK-1-1+). White triangles indicate the mock infection (0 dpi, n=6). Blue and red triangles indicate the infection dose of 2×10^3 $TCID_{50}$ (2, 4 and 7 dpi, n=6) and 2×10^4 $TCID_{50}$ (2 dpi, n=6. 4 dpi, n=5), respectively. Data are presented as the mean ± SEM. Statistical analyses were performed using Kruskal-Wallis one-way ANOVA followed by Dunn's multiple comparison test.

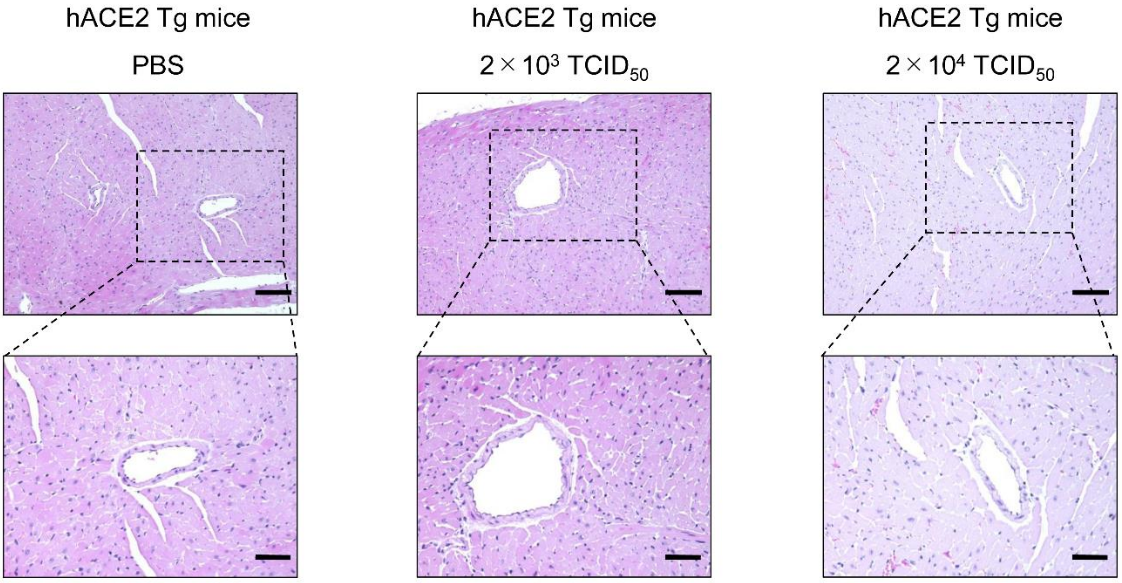

Supplemental Figure 8. Immunohistochemical analyses of hearts infected CAG-hACE2 mice. H&E staining of representative images are shown in the heart tissues of uninfected (left panel), deceased mice infected with 2×10^3 $TCID_{50}$ (center panel) and 2×10^4 $TCID_{50}$ (right panel) SARS-CoV-2. Bars indicates 100 µm (Upper images) and 50 µm (Bottom images).

Supplemental Table 1. Comparison of mouse model infected with SARS-CoV-2

Mouse model	Advantages/ Limitations	Route of infection	Lethal dose of SARS-CoV-2	Clinical signs	Histopathology	viral RNA	Immune response	Reference
CAG-hACE2 mouse	✓ Highly susceptible for SARS CoV-2<br>✓ Suitable for evaluating vaccine and therapeutics by lethality	Intratracheal	$10^3 \sim 10^4$ $TCID_{50}$	Weight loss and pneumonia	✓ Inflammatory cell infiltration<br>✓ Alveolar wall thickening<br>✓ Congestion and/or hemorrhage	Mainly lung, and brain	✓ Elevated cytokine and chemokine<br>✓ Lymphopenia (PBMC)<br>✓ Elevated neutrophil (PBMC)	-
		Intranasal	$10^2 \sim 10^4$ $TCID_{50}$	Weight loss	N/A	N/A	N/A	
K18-hACE2 mouse	✓ Susceptible for SARS CoV-2<br>✓ Suitable for evaluating vaccine and therapeutics by lethality	Intranasal	$10^4 \sim 10^5$ $TCID_{50}$	Weight loss and pneumonia	✓ Inflammatory cell infiltration<br>✓ Interstitial pneumonia<br>✓ Alveolar wall thickening	Lung, heart, brain, kidney, spleen, intestine, stomach	✓ Elevated cytokine and chemokine<br>✓ Elevated CD11b+ DCs (Lung)<br>✓ Decreased monocyte (Lung)	Bao et al., (2020)<br>Winkler et al., (2020)<br>Yinda et al., (2020)<br>Oladunni et al., (2020)
HFH4-hACE2 mouse	✓ Susceptible for SARS CoV-2<br>✓ Suitable for evaluating vaccine and therapeutics by lethality	Intranasal	$10^4 \sim 10^5$ $TCID_{50}$	Weight loss	✓ Interstitial pneumonia<br>✓ Inflammatory cell infiltration<br>✓ Hyaline membranes	Lung, heart, eye and brain	✓ Elevated neutrophil (PBMC)	Jiang et al., (2020)
Knock-in mouse	✓ Mild symptoms<br>✓ Regulated hACE2 expression by endogenous mACE2 promoter	Intranasal	N/A	N/A	✓ Inflammatory cell infiltration<br>✓ Alveolar wall thickening	Lung, trachea and brain	✓ Elevated cytokine and chemokine	Sun et al., (2020)
		Intratracheal	N/A	Congestion and edema of lung	✓ Neutrophil infiltration<br>✓ Hyaline membranes-like structure	Lung	✓ Elevated cytokine and chemokine	Hung et al., (2021)
Adeno virus vector-driven hACE2 mouse	✓ Applicable for all mice including disease model mice<br>✓ Mild symptoms	Intranasal	N/A	Weight loss	✓ Inflammatory cell infiltration	Lung, heart, and spleen	✓ Elevated cytokine and chemokine	Hassan et al., (2020)
Adeno-associated virus vector-driven hACE2 mouse	✓ Applicable for all mice including disease model mice<br>✓ Mild symptoms	Intranasal	N/A	N/A	✓ Perivenular inflammation<br>✓ Inflammatory cell infiltration	Lung	✓ Elevated macrophage, monocyte,<br>✓ T-cell, and NK cell (Lung)	Israelow et al., (2021)
mouse adopted SARS-CoV-2	✓ Structurally difference of S protein comparing with conventional S protein<br>✓ Mild symptoms	Intranasal	N/A	N/A	✓ Alveolar damage, focal exudation and hemorrhage<br>✓ Inflammatory cell infiltration	Lung, trachea, liver, spleen and heart	✓ Elevated cytokine and chemokine	Gu et al., (2021)

Supplemental Table 2. RT-qPCR primers for cytokine and chemokine mRNA measurements.

Genes	5'-Sense-3'	3'-Antisense-5'
β-Actin	ATGCAGAAGGAGATTACTGCTCTG	ATCTGCTGGAAGGTGGACAGTGA
Il-6	GCCAGAGTCCTTCAGAGAGATACAG	ACTCCTTCTGTGACTCCAGCTTATCT
Il-1b	GGGCCTCAAAGGAAAGAATCTATACC	GACTTCTATCTTGTTGAAGACAAACCG
Tnf-α	TGTCTACTGAACTTCGGGGTGA	GCTACAGGCTTGTCACTCGAATTT
Ifn-β	AGCAAGAGGAAAGATTGACGTGG	AAAGTTCCTGAAGATCTCTGCTCG
Ifn-γ	GGATGCATTCATGAGTATTGC	ACTCCTTTTCCGCTTCCTGA
Ccl2	TGAGTAGGCTGGAGAGCTACAA	TATGTCTGGACCCATTCCTTCTT
Ccl4	CTTCACAGAAGCTTTGTGATGG	ATGTACTCAGTGACCCAGGGCT
Ccl12	CCAGTCACGTGCTGTTATAATGTTGTT	ACAGATCTCCTTATCCAGTATGGTCCT
Cxcl1	ATGGCTGGGATTCACCTCAA	GAGCTTCAGGGTCAAGGCAA
Cxcl10	GCCGTCATTTTCTGCCTCAT	GCTTCCCTATGGCCCTCATT

Supplemental Table 3. Antibodies using FACS analyses.

Antibody	Clone	Catalog No.	Company	Dilution
PerCP-Cy5.5 Hamster Anti-Mouse CD3e	145-2C11	551163	BD Bioscience	1:100
BUV496 Rat Anti-Mouse CD4	GK1.5	612952	BD Bioscience	1:100
BUV805 Rat Anti-Mouse CD8a	53-6.7	612898	BD Bioscience	1:100
BV480 Rat Anti-CD11b	M1/70	566117	BD Bioscience	1:100
BB515 Armenian Hamster Anti-Mouse CD11c	N418	565586	BD Bioscience	1:100
BV650 Rat Anti-Mouse CD19	1D3	563235	BD Bioscience	1:100
PE-CF594 Rat Anti-Mouse CD49b	DX5	562453	BD Bioscience	1:100
PE anti-mouse CD200R3	Ba13	142206	BioLegend	1:100
PE-Cy7 conjugated Rat Anti-Mouse Ly-6C	AL-21	560593	BD Bioscience	1:100
BUV395 Rat Anti-Mouse Ly-6G	1A8	563978	BD Bioscience	1:100
Alexa Fluor® 647 Rat Anti-Mouse I-A/I-E	M5/114.15.2	562367	BD Bioscience	1:100
BV421 Rat Anti-Mouse Siglec-F	E50-2440	565934	BD Bioscience	1:100
BV786 Mouse Anti-Mouse NK-1.1	PK136	740853	BD Bioscience	1:100

Supplemental Methods

Intranasal infection of SARS-CoV-2. Mice were assigned randomly to three groups in CAG-hACE2 mice to assess hACE2 Tg + 2×10^2 $TCID_{50}$ (n=6), hACE2 Tg + 2×10^3 $TCID_{50}$ (n=6), and hACE2 Tg + 2×10^4 $TCID_{50}$ (n=6). CAG-hACE2 mice were intranasally infected with SARS-CoV-2 stock virus at a dosage of 2×10^2 $TCID_{50}$/10 μL, 2×10^3 $TCID_{50}$/10 μL and 2×10^4 $TCID_{50}$/10 μL (5 μL/nostril). Infected mice were recorded daily for body weight and survival. Mice that were clearly emaciated were euthanized after recording their body weight and were considered dead.

Macroscopic and Histological Evaluations. Lung and heart samples were collected from SARS-CoV-2 infected and uninfected mice. These organs were immersed in 10% formalin for 24 h, embedded in paraffin, and cut into 2 and sections onto a slide glass (Matsunami Glass, Osaka, Japan). Tissue sections were stained with hematoxylin and eosin (H&E) and observed using a BZ-9000 microscope (HS All-in-One Fluorescence Microscope; Keyence, Osaka, Japan) at ×200, and ×400 magnification.

Flow cytometry. CAG-hACE2 mice were sacrificed at 0, 2, 4, and 7 dpi to collect their blood and spleen. Sample preparation as a below. Splenocytes were collected using C-

tube (gentleMACS™ C Tubes, Cat# 130-093-237, Miltenyi Biotec Inc., Bergisch Gladbach, Germany) and hemolyzed using lysing solution (Pharm Lyse™; Cat# 555899, BD Biosciences, NJ, USA). Blood was hemolyzed using lysing solution to collect Peripheral Blood Mononuclear Cells (PBMCs). Splenocytes and PBMCs were stained by Fixable Viability Stain 780 (Cat# 565388, BD Biosciences) to separate live cells and dead cells followed by blocking with Mouse BD Fc Block™ (Purified Rat Anti-Mouse CD16/CD32; Cat# 553142, BD Biosciences). After blocking, PBMCs were stained with the antibodies (supplementally Table 3) in Brilliant Stain Buffer Plus (Cat# 566385, BD Biosciences). After staining, cells were fixed in 0.5% PFA/FACS buffer (4% Paraformaldehyde Phosphate Buffer Solution, Cat# 163-20145, FUJIFILM Wako Pure Chemical Corporation, Tokyo, Japan.) and SARS-CoV-2 was inactivated by the buffer. Flow cytometric analysis of PBMCs was performed using a LSR Fortessa™ X-20 (BD), and data were analyzed with Diva software (BD Biosciences) and FlowJo software (FlowJo™, V10, BD).

Statistics

Data are presented as the mean ± SEM. Statistical analyses were performed using GraphPad Prism 7.0. (GraphPad Software, La Jolla, CA, USA). Kruskal-Wallis one-way

ANOVA followed by Dunn’s multiple comparison test was used for the flow cytometry analysis of PBMC and splenocyte. *Statistical significance was set at P*<0.05.
